# Characterization and decontamination of background noise in droplet-based single-cell protein expression data with DecontPro

**DOI:** 10.1093/nar/gkad1032

**Published:** 2023-11-17

**Authors:** Yuan Yin, Masanao Yajima, Joshua D Campbell

**Affiliations:** Section of Computational Biomedicine, Department of Medicine, Boston University School of Medicine, Boston, MA 02118, USA; Department of Mathematics and Statistics, Boston University, Boston, MA 02115, USA; Section of Computational Biomedicine, Department of Medicine, Boston University School of Medicine, Boston, MA 02118, USA

## Abstract

Assays such as CITE-seq can measure the abundance of cell surface proteins on individual cells using antibody derived tags (ADTs). However, many ADTs have high levels of background noise that can obfuscate down-stream analyses. In an exploratory analysis of PBMC datasets, we find that some droplets that were originally called ‘empty’ due to low levels of RNA contained high levels of ADTs and likely corresponded to neutrophils. We identified a novel type of artifact in the empty droplets called a ‘spongelet’ which has medium levels of ADT expression and is distinct from ambient noise. ADT expression levels in the spongelets correlate to ADT expression levels in the background peak of true cells in several datasets suggesting that they can contribute to background noise along with ambient ADTs. We then developed DecontPro, a novel Bayesian hierarchical model that can decontaminate ADT data by estimating and removing contamination from these sources. DecontPro outperforms other decontamination tools in removing aberrantly expressed ADTs while retaining native ADTs and in improving clustering specificity. Overall, these results suggest that identification of empty drops should be performed separately for RNA and ADT data and that DecontPro can be incorporated into CITE-seq workflows to improve the quality of downstream analyses.

## Graphical Abstract

**Graphical Abstract:**
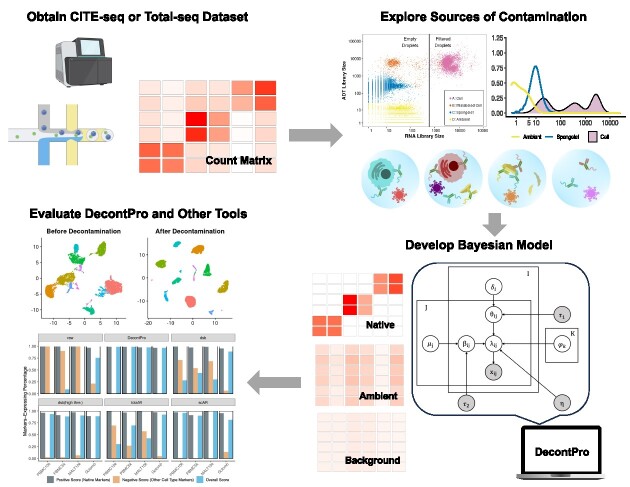

## Introduction

Cellular indexing of transcriptomes and epitopes by sequencing (CITE-seq) is an assay that can quantify the abundance of RNA transcripts as well as cell surface proteins on individual cells (1). Antibody-derived tags (ADTs) that bind to cell surface proteins are measured during sequencing to produce an ADT count matrix that quantifies the levels of these surface proteins. Information on the abundance of proteins complements single-cell RNA-seq (scRNA-seq) data and improves the ability to describe cell type and cell states with functional annotations (2–4). Other variants of CITE-seq have been developed which can measure proteins in other settings such as CRISPR perturbations, single-cell ATAC-seq or spatially resolved expression (5–7).

Previous studies have noted that individual ADTs often have a multimodal distribution including one lower ‘background’ peak and one or more higher peaks attributed to the true signal from the cells. The lower background peak has been attributed to noise from non-specific binding of antibodies (8). Algorithms such as TotalVI (9) have tried to leverage the multi-modal nature to identify and remove the lower background peak for each ADT. Other approaches try to measure and remove background levels using ‘spike-in’ reference cells from another species such as mouse (1). Utilizing spike-ins adds extra complexity to the experimental design and assumes that rate of contamination in the reference cells will be the same for the cells of interest in the dataset. Additionally, none of these approaches dissect out different sources of contamination within each cell.

Contamination from various sources can contribute to poor-quality data in single-cell assays. In scRNA-seq data, ambient RNA from the cell suspension can be counted along with a cell’s native RNA and result in contamination of gene markers between cell types (10,11). Ambient contamination may also occur in CITE-seq data as the methods for generating CITE-seq data also rely on microfluidic droplet-based devices. Two computational methods, dsb (12) and scAR (13), have been proposed that use the ADT expression profiles of the ‘empty droplets’, i.e. droplets without a true cell to estimate and remove the noise from ambient material. However, these methods treat the empty droplets as a single source of noise. Furthermore, reliance on the empty droplets data may limit their application in cases where the empty droplet matrix is not available.

In this study, we analyzed four PBMC CITE-seq datasets and showed that there are at least four different types of droplets including (i) droplets containing true cells with high RNA and high ADT content, (ii) droplets with low RNA content and high ADT content that are mislabeled as ‘empty droplets’, (iii) droplets containing low levels ADTs matching ambient distributions and (iv) droplets containing medium levels of ADTs with non-specific distributions, which we denote as ‘spongelets’. We show that the ADT expression profiles of spongelets are highly correlated with the expression profiles of the background peak in true cells and likely contribute to contamination along with ambient ADTs. Based on these results, we developed a novel Bayesian hierarchical model called DecontPro (**Decont**amination of **Pro**tein expression data) that removes the background peak by estimating ambient contamination as well as contamination derived from other sources such as spongelets or non-specific binding. When applied to different ADT datasets, DecontPro was able to preserve the expression of native markers in known cell types while removing contamination from the non-native markers. DecontPro outperformed other tools in removing non-native markers and in improving downstream clustering in several benchmarking datasets. Finally, we show that DecontPro can increase the specificity of PD-1 expression in activated T and B-cells.

## Materials and methods

### Exploratory droplet analyses

To characterize different sources of contamination of the ADT datasets, we performed an exploratory analysis of four datasets. We utilized the PBMC 10k dataset containing cells from a healthy donor stained with 17 Total-Seq-B antibodies (7865 filtered droplets; 6 794 880 raw droplets), the PBMC 5k dataset containing cells from a healthy donor stained with 31 Total-Seq-B antibodies (5527 filtered droplets; 6 794 880 raw droplets), the MALT 10k dataset containing dissociated extranodal marginal zone B-cell cells from a tumor stained with 17 Total-Seq-B antibodies (8412 filtered droplets; 6 794 880 raw droplets), and the Golomb dataset containing cells from brain samples of mice with antibiotics induced gut microbiota depletion (30 569 filtered droplets; 13 589 760 raw droplets). For the Golomb dataset, a panel of 31 antibodies was used for staining the samples of 3 young mice and 3 aged mice, and the samples were pooled together using hashtag oligo (14). Droplets with zero total counts for both ADT and RNA were excluded. The library sizes for ADT and RNA were calculated by summing all the ADT and RNA counts in each droplet. The droplets present in the raw matrix but not the filtered matrix produced by CellRanger were considered empty droplets. The remaining droplets from the raw matrix were clustered with using *k*-means with the number of clusters set to 3. We calculated the normalized ADT profiles of the spongelet and ambient clusters by summing counts of each ADT across droplets in that cluster and dividing by the total ADT count in that cluster. The background profile for each ADT was calculated by summing ADT counts that fell into the background peak and dividing by the total ADT count.

### The DecontPro statistical model

Based on the results of the exploratory analysis we detailed in the results section ‘Different Contamination Profiles Contribute to CITE-seq Data’, we developed a Bayesian hierarchical model to decontaminate ADT data that estimates and removes counts from both ambient and other background sources. The model uses cell-containing count matrix $X$ as input. For a droplet $i$ containing a cell of type ${k}_i$ having the library size ${L}_i$, we assume the ${j}^{th}$ ADT count ${x}_{ij}$ is a realization from a Poisson distribution with rate parameter ${\lambda }_{ij}$:


\begin{eqnarray*}{x}_{ij}|L\sim Poisson({\lambda}_{ij}{L}_{i})\end{eqnarray*}

When there is a total of $I$ droplets, $J$ ADTs and $K$ cell types, the count matrix can be organized into an $I$ by $J$ matrix $X = [ {{x}_{ij}} ]$ and a corresponding cell type indicator vector $k = [ {{k}_i} ]$. Based on our exploratory analyses, we further assume that the counts ${x}_{ij}$ are contributed by cell native ADT, ambient contaminating ADT, and other sources of background contamination such as spongelets. Therefore, the overall expected rate of counts for an ADT in a cell (${\lambda }_{ij}$) can be described as a sum of three components:


\begin{eqnarray*}{\lambda }_{ij} = \left( {1 - {\beta }_{ij}} \right)*{\theta }_{ij}*{\eta }_j + \left( {1 - {\beta }_{ij}} \right)*\left( {1 - {\theta }_{ij}} \right)*{\phi }_{{k}_ij} + {\beta }_{ij}\end{eqnarray*}

where ${\phi }_{{k}_ij}$ is the normalized rate of counts for ADT $j$ in cell population ${k}_i$. ${\phi }_{{k}_ij}$ is a multinomial distribution representing the percentage of native expression attributed to ADT $j$. ${\eta }_j$ is the normalized rate of counts due to the ambient source for ADT $j$. It is a multinomial distribution representing the percentage of ambient expression attributed to ADT $j$. ${\theta }_{ij}$ is the proportion of ambient material for ADT $j$ in cell $i$, and ${\beta }_{ij}$ is the normalized rate of contamination from all other background sources including spongelets and non-specific binding for ADT $j$ in droplet $i$.

We assume that some level of general background (${\beta }_{ij}$) will be present for each ADT in each cell and that the rates of native or ambient ADTs can be quantified in each droplet after subtracting out the background rate ($1 - {\beta }_{ij}$). More specifically, the normalized rate of ambient ADTs can be expressed as the expected proportion of counts coming from the ambient distribution times the level of ambient contamination in that cell after excluding the general background rate: $( {1 - {\beta }_{ij}} )*{\theta }_{ij}*{\eta }_j$. The rate of true native ADT counts for a given cell can be expressed as the expected number of counts from that cell type after excluding the proportion of counts from both ambient contamination and background noise: $( {1 - {\beta }_{ij}} )*( {1 - {\theta }_{ij}} )*{\phi }_{{k}_ij}$.

We put prior distributions on the rates of ambient contamination for all ADTs in a droplet and the background contamination for each ADT across all cells. ${\beta }_{ij}$ is drawn from a truncated normal parameterized by mean ${\mu }_j$ and bounded by (0, 0.5) while ${\theta }_{ij}$ is drawn from a truncated normal parameterized by mean ${\delta }_i$ and bounded by (0, 1):


\begin{eqnarray*}{\beta }_{ij}\sim\,Truncated\ Normal\left({\mu}_{j},{\tau}_{2} \right)\end{eqnarray*}

\begin{eqnarray*}{\theta }_{ij}\sim\,Truncated\ Normal\left({\delta}_i,{\tau}_{1} \right)\end{eqnarray*}

**Figure 1.**
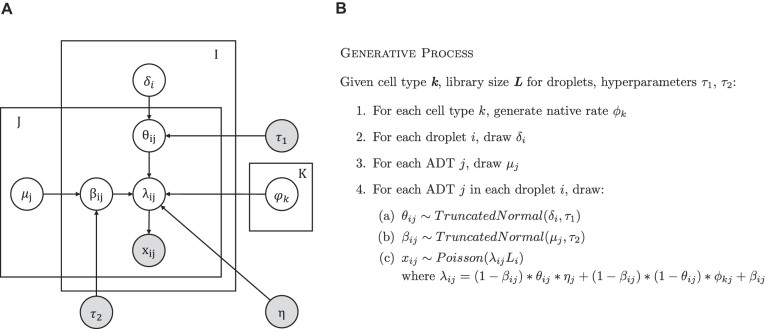
Summary of the DecontPro model. (**A**) Plate diagram for the DecontPro model. (**B**) The generative process of the DecontPro model.

We set the upper bound of $\beta$ to 0.5, as we assume that a cell has <50% of its counts contributed by background signal. Prior parameter ${\mu }_j$ for ${\beta }_{ij}$ represents the average background contamination for ADT $j$ while prior parameter ${\delta }_i$ for ${\theta }_{ij}$ represents the average ambient contamination level for droplet $i$.$\ {\tau }_1$ and ${\tau }_2$ are hyperparameters that control how strongly individual contamination rates can deviate from the prior mean rates of ${\delta }_i$ and ${\mu }_j$, respectively. The plate diagram for the model is shown in Figure 1.

After inference on the model, the actual counts ${x}_{ij}$ were divided into three portions, the native, the ambient and the background, based on each portion’s weight in the summation in the Poisson’s rate parameter.


\begin{eqnarray*}&&{x}_{i{j}_{native}} \\ &&={x}_{ij}*\frac{{\left( {1 - {\beta }_{ij}} \right)*\left( {1 - {\theta }_{ij}} \right)*{\phi }_{{k}_ij}}}{{\left( {1 - {\beta }_{ij}} \right)*{\theta }_{ij}*{\eta }_j + \left( {1 - {\beta }_{ij}} \right)*\left( {1 - {\theta }_{ij}} \right)*{\phi }_{{k}_ij} + {\beta }_{ij}}}\end{eqnarray*}

\begin{eqnarray*}&&{x}_{i{j}_{ambient}} \\ &&= {x}_{ij}*\frac{{\left( {1 - {\beta }_{ij}} \right)*{\theta }_{ij}*{\eta }_j}}{{\left( {1 - {\beta }_{ij}} \right)*{\theta }_{ij}*{\eta }_j + \left( {1 - {\beta }_{ij}} \right)*\left( {1 - {\theta }_{ij}} \right)*{\phi }_{{k}_ij} + {\beta }_{ij}}}\end{eqnarray*}

\begin{eqnarray*}&&{x}_{i{j}_{background}} \\ &&= {x}_{ij}*\frac{{{\beta }_{ij}}}{{\left( {1 - {\beta }_{ij}} \right)*{\theta }_{ij}*{\eta }_j + \left( {1 - {\beta }_{ij}} \right)*\left( {1 - {\theta }_{ij}} \right)*{\phi }_{{k}_ij} + {\beta }_{ij}}}\end{eqnarray*}

### Application of DecontPro to ADT datasets

We implemented this model in the decontX R package v0.99.3 (see Availability of Data and Materials) and applied it to the benchmarking datasets. Datasets were preprocessed by removing HTO tags and isotypes, filtering out cell droplets with top and bottom one percent of ADT and RNA total library sizes, and droplets with 15% or higher mitochondrial gene counts. As inputs to the software, cell clusters were generated using the Seurat package v4.2.0. For clustering we used RunPCA with npcs = 10 except for Golomb dataset where we used npcs = 20; FindNeighbors with dims = 1:10; FindClusters with res = 0.2 except for MALT10k and Golomb dataset where we used res = 0.3. The prior parameters were set to ${\tau }_1$(PBMC 10k: 2e-5, PBMC 5k: 5e-5, MALT 10k: 4e-5, Golomb: 2e-5) and ${\tau }_2$(PBMC 10k: 2e-6, PBMC 5k: 5e-6, MALT 10k: 2e-6, Golomb: 2e-6). For inference on the model, we used Automatic Differentiation Variational Inference (ADVI) implemented in Stan. ${\theta }_{ij}$ was initialized 1e-4 and ${\beta }_{ij}$ was initialized 1e-2. The max number of iterations were set to 50 000.

### Benchmarking of other decontamination tools

We benchmarked the performance of DecontPro against three other decontamination methods, dsb (12), totalVI (9) and scAR (13). Dsb uses empty droplets to correct ambient noise and uses model-fitting and data transformation to identify and remove technical noise. We used filters of log scaled protein counts between 1.5 and 3 and log scaled RNA counts <2.5 to identify empty droplets. We used DSBNormalizeProtein with use.isotype.control = TRUE for PBMC10k, PBMC5k and MALT10k which have isotypes. TotalVI finds a joint representation of both RNA and protein data of cells, which is decoded into native and noise estimation of proteins. We used scanpy.pp.normalize_total with target_sum = 1e4; scanpy.pp.highly_variable_genes with n_top_genes = 4000, flavor=‘seurat_v3’, subset = True, and layer=‘counts’ while setting up data objects for model training and SCVI. get_normalized_expression with n_samples = 25 when retrieving decontaminated counts from the trained model. ScAR is a deep learning method where empty droplets were used to estimate ambient noise. For scAR, we used droplets with total RNA counts <100 and ADT counts <25 as the empty droplets. We set up the model with feature_type=‘ADT’, count_model=‘binomial’ and model.train with epochs = 80, batch_size = 64 when training the model. All the cell droplets were preprocessed and filtered the same as they were for DecontPro before applying each method, which we described in previous section. The links to the code used to run these methods can be found in the ‘Availability of Data and Materials’ section.

### Assessment of benchmarking results

The four datasets in our exploratory analyses also were used for benchmarking. First, we used the change in mean silhouette width before and after decontamination to assess the ability of tools to produce more distinct clusters. Cluster labels were generated using the default workflow in the Seurat package for ADT data. Second, we developed three metrics to assess the ability of each tool to remove contaminating cell type markers from non-native cell types while maintaining the expression of true markers in each cell type. For each dataset, we annotated ADTs that we expected to be expressed in each cell cluster based on prior biological knowledge and ADTs not expected to be expressed in each cluster. For example, in PBMC datasets we expected CD3 to be expressed in T-cells but not in B-cells while CD14 were expected to be expressed in CD14 + monocytes but not in T-cells populations. The cell clusters and ADT markers used to calculate the scores are listed in Supplementary Table S1. We defined the Positive Score as the percentage of cells that have detectable levels of true, native cell type markers after decontamination while the Negative Score was defined as the percentage of cells that have detectable levels of contaminating, non-native markers. The threshold for detection was set to 1 for all datasets. For dsb, we also assessed an additional higher detection threshold of 5, reported as ‘dsb (high threshold)’, to account for the fact that dsb results are normalized and thus are on a different scale compared to outputs from other tools. The Combined Score was defined as the Positive Score minus the Negative Score. For annotation of cell clusters, we find differentially expressed ADTs and genes using FindAllMarkers of Seurat package with min.pct = 0.25 and only.pos = TRUE (Supplementary Figure S1).

## Results

### Different contamination profiles contribute to CITE-seq data

**Figure 2.**
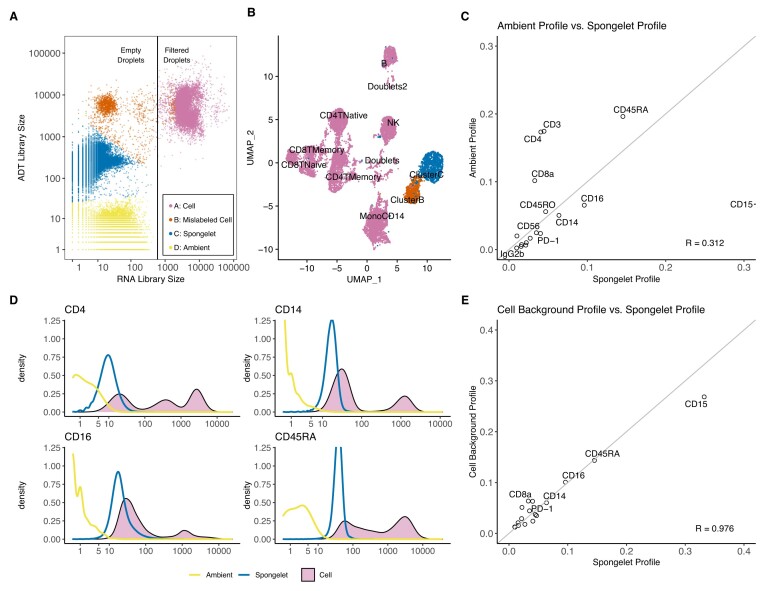
Analysis of ADT droplets in the PBMC 10K dataset. (**A**) Among all droplets, only the cluster with high RNA-seq library size were filtered as cell droplets by the 10x Genomics software, while we observed clusters with varying levels of ADT library sizes among the droplets labeled as empty. We identified three empty droplet clusters, and called them clusters B, C and D. (**B**) A thousand droplets sampled from clusters C and D each were combined with Cell droplets for clustering using Seurat workflow. Distinct clusters were observed. (**C**) The empirical distribution built using the droplets from cluster C (spongelet) and D (ambient) showed poor correlation. (**D**) Density plots of droplets from the cluster C were close to the background peak of the cell density plot. (**E**) We manually curate the background peaks of the cell density (Supplementary Figures S15–S17) and normalize them into a multinomial distribution. The distribution was found to be highly correlated with the spongelet cluster empirical distribution.

We performed an exploratory analysis of CITE-seq and Total-seq datasets to understand heterogeneity among the droplets and to characterize different sources of contamination. We first analyzed a public dataset containing peripheral blood mononuclear cells from a healthy donor from 10x Genomics (PBMC 10K). Four distinct clusters of droplets were observed when comparing the total UMI counts of ADTs to total UMI counts of RNAs in each droplet (Figure 2A). Cluster A had high counts for both RNA and ADTs and were called true cells by Cell Ranger (*n* = 7864 droplets). Clusters B–D were called empty droplets by Cell Ranger and had low RNA counts with varying levels of ADT counts. Cluster D contained droplets with low levels of both total RNA and total ADT counts (*n* = 145 322 droplets). The average profile of the droplets from this cluster was highly correlated with the average profile of droplets from the Cell cluster for both RNA and ADTs for most datasets (*R* > 0.950) except for the Golomb dataset (*R* = 0.769, Supplementary Figure S2) demonstrating that these droplets likely contain only ambient material (9).

Cluster B had an average of 5168 ADTs counts per droplet (*n* = 1406 droplets) while cluster C had an average of 268 ADTs (*n* = 70 157 droplets). To understand the ADT profiles in these clusters, we randomly sampled a thousand droplets from each of these two clusters and analyzed them with the droplets containing cells from cluster A using the standard Seurat clustering workflow (15). Droplets from the clusters B and C formed their own distinct clusters (Figure 2B). Cluster B had significantly higher levels of CD15 and CD16 compared to other populations, but few differentially expressed genes in the RNA data (Supplementary Figure S3). This ‘mislabeled cell’ cluster likely represents neutrophils which are prevalent in white blood cells but have low RNA content (16,17) and suggests that viable cells with low RNA content may be readily characterized by ADT expression. Therefore, filtering of empty droplets should be performed separately for ADT and RNA data. In contrast to the mislabeled cell cluster B, cluster C did not show strong enrichment for any ADTs. The average profile of droplets in cluster C was not highly correlated to the average profile of the ambient cluster D (*R* = 0.312, Figure 2C), confirming that the source of this cluster is not solely related to ambient ADTs. We assigned the name ‘spongelets’ to the droplets in cluster C given that they contain medium levels of ADTs that do not have enrichment in specific cell types.

The distribution of individual ADT expression in true cells is often multi-modal and contains more than one peak. For example, cells in cluster A have distributions of CD14, CD16 and CD45RA as bi-modal and the distribution of CD4 as tri-modal (Figure 2D). The lower peak of the multi-modal densities has previously been characterized as background signals from non-specific binding of antibodies (8). Interestingly, the density of ADTs in the spongelet cluster largely overlapped with the density of the lower peak in the cluster A (Figure 2D and Supplementary Figure S4). This overlap was also observed in the three other ADT datasets (Supplementary Figures S5–S8). The average percentage of each ADT in the lower peak of cluster A was highly correlated with the average percentage of each ADT in cluster C in the PBMC 10K dataset (*R* = 0.976, Figure 2E), and to a lesser degree in two other datasets (*R* = 0.720 and *R* = 0.641, Supplementary Figure S9). The low-level background expression indicated by the lower peak is also manifested after normalizing Cell droplets by their library sizes and classifying them by their cell types (Supplementary Figure S10–S12). Low peaks of ADTs were observed in cell types that are not supposed to express the ADTs. Interestingly, the density showing the background peak for each ADT has a consistent mean across cell types. Overall, these findings suggest an association between the ADT expression profiles of spongelets and the lower background peaks observed in true cells.

### A novel model for estimating and removing decontamination

**Figure 3.**
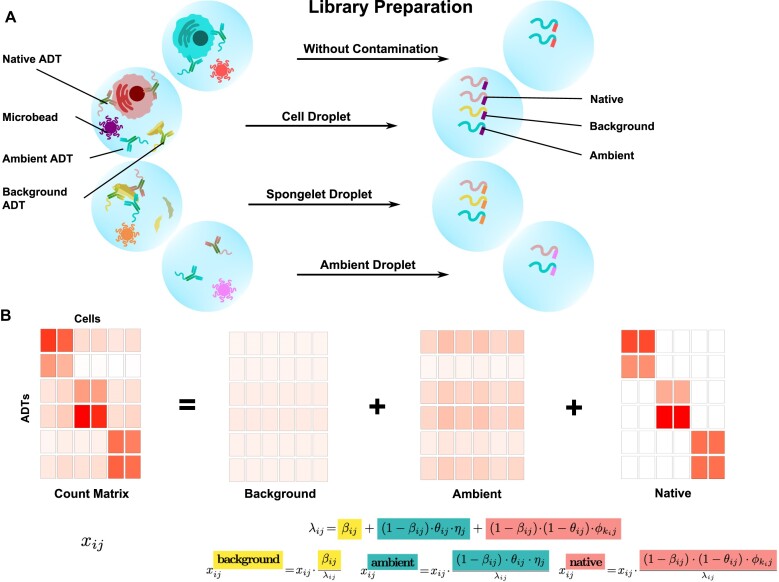
DecontPro can estimate and remove contamination from multiple sources in CITE-seq data. (**A**) Single cells and ADTs are isolated in droplet compartments. Spongelet droplets contain some non-specific distribution and an intermediary level of ADTs, while ambient droplets have a low level of ADTs attributed to the ambient material from the cell suspension. Native ADTs refer to those that bind to their target cell surface proteins in the appropriate cell population. Background ADTs represent those from contamination sources other than ambient such as spongelets or non-specific binding of ADTs. These different sources of ADTs can be present and quantified together in each droplet during library preparation. (**B**) The DecontPro model can deconvolute an ADT count matrix into a background matrix, an ambient matrix and a native matrix along with the proportion of counts attributed to each source in each cell. The native matrix can be used in downstream analyses.

We next sought to build a deconvolution algorithm that can estimate and remove contamination from the ambient material as well as any other sources contributing to the background including spongelets and other non-specific binding. We assume that each cell is a mixture of three sources: (i) ADTs from the native cell, (ii) ADTs from the ambient material present in the cell suspension and (iii) ADTs from any contamination source that contributes to a lower non-specific background peak (Figure 3A). The model takes a cell-containing count matrix as input and decomposes it into three matrices, each containing a proportion of the original counts as an estimate of the contribution from the three sources (Figure 3B, Materials and methods).

When the raw matrix with empty droplets is available, the ambient profile $\eta$ can be estimated using the empirical distribution of the ambient droplets (i.e. droplets with low expression of ADTs). If the raw matrix is not provided, the ambient profile $\eta$ for a dataset can be estimated by calculating the average of the ADT across filtered cell droplets. This is due to the fact that the average cell profile is highly correlated to the empirical distribution of the ambient droplets in the majority of datasets. For example, we observed this pattern in three ADT datasets (PBMC 10k: *R* = 0.977, PBMC 5k: *R* = 0.991, MALT 10k: *R* = 0.968; Supplementary Figure S2). The correlation is less strong for one dataset (Golomb: *R* = 0.769), primarily due to an outlier ADT Ly6C. Ly6C had uniformly high level of expression across droplets while other highly expressed ADTs were more localized to specific clusters (Supplementary Figure S13). We use the variational inference framework provided by Stan to estimate the remaining parameters and deconvolute the ADT count matrix into the native, ambient and background matrices for downstream analysis (Figure 3B). Detailed description of the model can be found in the Materials and methods section.

### Decontamination of PBMCs

**Figure 4.**
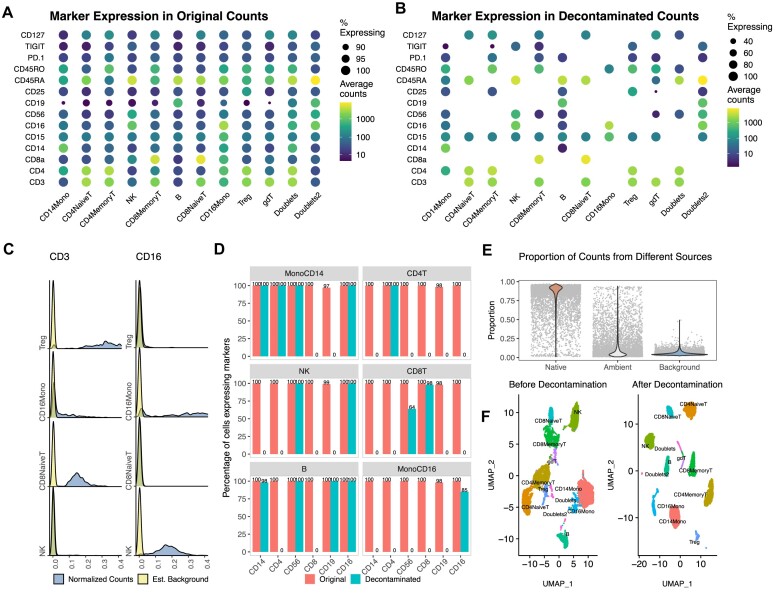
Decontamination of the PBMC 10k dataset with DecontPro. (**A**) Expression profiles of ADTs in cell populations before decontamination. All ADTs were expressed in each cell population to some degree. (**B**) After decontamination with DecontPro, aberrantly expressed ADTs were removed or greatly reduced in non-native cell populations. (**C**) The density of normalized ADT expression for CD3 and CD16 in selected cell populations. The model estimated background was superimposed on to the density plot. (**D**) Percentage of cells expressing known cell type markers before and after decontamination in different cell populations. Markers with a count greater than one were considered expressed in a cell. (**E**) The DecontPro model estimates the proportion of counts coming from native, ambient and background sources in each droplet. The median percentage of native, ambient and background counts was 89.5%, 4.4% and 5.5%, respectively. (**F**) Using decontaminated counts improved separation of clusters on a UMAP.

To demonstrate the ability of our method to improve data quality, we applied DecontPro to the PBMC 10k dataset from 10x Genomics and compared the distributions of counts before and after decontamination. In the original count matrix, all ADTs were expressed to some degree in nearly every cell cluster (Figure 4A). After decontamination, known markers for cell types remained highly expressed (Figure 4B). For example, CD19 is a B-cell marker with an average expression of 668.46 in the B-cell cluster and a low average expression of 13.68 across other cell types in the original count matrix. After decontamination the only clusters retaining expression of CD19 were the B-cells and doublets. Similarly, T-cell marker CD3 had remaining counts only in the T-cell clusters and a doublet cluster.

For most markers, the density of the estimated background largely overlapped with the lower peak across cell clusters. For example, the lower levels of CD3 were estimated to be largely background and removed in the CD16 Monocyte and NK clusters (Figure 4C). Similarly, for CD16 the lower levels of expression were estimated to be largely background in Naïve CD8 T-cells and T-regs clusters. As expected, native ADTs in their corresponding clusters were expressed at a higher level, and hence had a higher peak in the normalized counts’ density, such as CD3 in CD8 Naïve T-cells cluster and T-regs cluster.

To systematically assess how well the algorithm performed in specificity in retaining native ADTs for clusters, we calculated the percentage of cells in each cluster expressing native marker ADTs before and after decontamination (Figure 4D). Markers and cell types included CD3 and CD4 for CD4 + T-cells, CD3 and CD8 for CD8 + T-cells, CD19 for B-cells, CD14 for CD14 + monocytes, CD16 for CD16 + monocytes, and CD56 for NK cells. In many cases, the algorithm was able to greatly reduce or completely remove aberrant counts in non-native cell types. For example, CD14 expression was removed from T-cells, CD19 was removed from T-cells, NK-cells, and monocytes, and CD3 was removed from B-cells and monocytes. In some cases, the reduction left some ADT values in unexpected cell clusters. The monocyte marker CD14 was still detected in a high percentage of B-cells after decontamination (98%). However, the overall level of CD14 in B-cells was still greatly reduced compared to the original ADT counts (Supplementary Figure S14).

DecontPro also estimates the percentage of counts contributed by the native, ambient and background signals (Figure 4E). As expected, the native counts took the highest percentage of libraries across droplets on average (median 89.5%, range: 1.0–97.1%), whereas the background counts were estimated at a consistently lower amount (median 4.4%, range: 2.0–49.2%). The percentage of ambient ADTs had a similar median to the percentage of background counts, but a much larger range (median 5.5%, range: 0.8–93.9%). Lastly, the clusters in the UMAP generated with the decontaminated counts were more separated compared to clusters in the UMAP generated with the original counts (Figure 4F).

### Benchmarking against other methods

**Figure 5.**
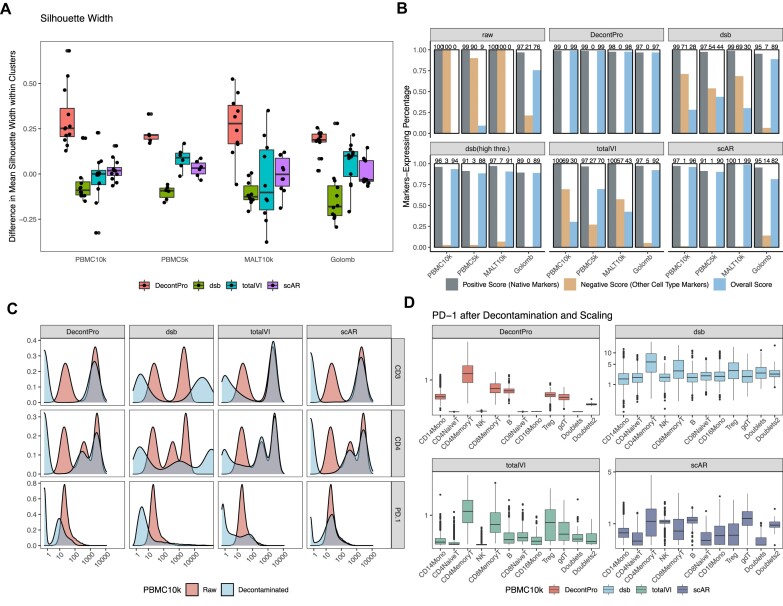
Benchmarking of ADT decontamination methods. We compared the ability of four algorithms to remove contamination from four datasets. The other methods include dsb, scAR and totalVI. (**A**) The average silhouette width for each cell cluster identified by Seurat was calculated before and after decontamination of ADT counts. A higher difference in silhouette widths indicates better improvement in cell similarity within that cluster. On average, DecontPro improved silhouette widths better than other methods for each dataset. (**B**) For each dataset, a pair of scores were calculated after applying each decontamination algorithm to ascertain the degree to which each algorithm maintains true expression while removing contamination. The ‘positive’ score was defined as the percentage of cells expressing native markers averaged across cell clusters. In contrast, the ‘negative’ score was defined as the percentage of cells expressing non-native markers averaged across cell clusters. For example, in the CD4 naïve T-cells cluster, CD4 and CD45RA were defined as the native markers, while CD8 and CD45RO were defined as the non-native markers. As dsb outputs normalized continuous counts after decontamination, we applied two different thresholds to determine which markers were detected in each cell. The overall score is the positive score minus the negative score. Overall, DecontPro performs the best in the overall score in all four datasets. (**C**) Density plot of CD3, CD4 and PD-1 before and after decontamination in the PBMC 10k dataset. The dsb output was exponentiated to allow for comparison with the uncorrected original count density. (**D**) Box plot of PD-1 level in cell clusters after decontamination and scaling PBMC 10k dataset.

We benchmarked DecontPro against three other decontamination algorithms applicable to CITE-seq datasets: dsb, scAR and totalVI. To compare how well decontamination improved clustering, we calculated the mean silhouette width of each cell cluster identified by Seurat before and after decontaminating using four public datasets. A higher mean silhouette width indicates higher similarities between cells within each cluster. Across all datasets, DecontPro showed the highest average improvement in silhouette widths after decontamination (Figure 5A). We next sought to understand how effectively various algorithms improved the specificity of marker expressions in cell clusters. Specifically, we wanted to measure if the expected markers in annotated clusters retained their expression, while the unexpected markers from other cell types were removed. We calculated two scores for each algorithm on each dataset. A positive score was calculated by finding the percentage of cells in a cluster that express the expected ‘native’ markers while a negative score calculated the percentage of cells in a cluster that express unexpected ‘non-native’ markers from other cell types. An effective decontamination algorithm will have a positive score close to 100 indicating that the expression of native markers was retained in their true cell population as well as a negative score close to 0 indicating that the expression of non-native markers from other cell types was successfully removed. The full list of annotated native markers for each cell type can be found in the Materials and methods section. As a reference, the uncorrected original count data had high positive scores in all datasets and high negative scores close to 100 for the PBMC 10k, PBMC 5k and MALT 10k datasets, indicating a high level of contamination in most cell types (Figure 5B). When using the difference between the positive score and the negative score as one overall score to measure the algorithm performance on a dataset, DecontPro had the highest scores in almost all dataset, indicating that it successfully retained the expected markers while removing the unexpected markers across datasets better than other tools (PBMC 10k: 99, PBMC 5k: 99, MALT 10k: 98, Golomb: 97). The only exception was scAR on the MALT10k dataset which obtained an overall score of 99. As dsb performs its own normalization and scaling, the decontaminated count matrix is not directly comparable with the original count matrix. We therefore used two different thresholds for a maker to be considered detected in a cell (dsb: 1; dsb high threshold: 5) and calculated the scores accordingly. The higher threshold for dsb produced better negative scores more similar to algorithms such as DecontPro and scAR, at the expense of decreasing the positive scores in each dataset. The second-best performing algorithm across datasets based on difference between positive and negative scores was scAR (PBMC 10k: 96, PBMC 5k: 90, MALT 10k: 99, Golomb: 82).

For the PBMC 10k dataset, the decontamination results for known cell type markers were mostly similar across algorithms. For antibodies with high expression, such as CD3 and CD4, all algorithms retained the higher peak while significantly reducing the lower background peak (Figure 5C). However, PD-1 was expressed at lower levels and demonstrated a noticeable difference in decontamination results between algorithms. In PBMCs, PD-1 is predominantly expressed in activated CD8 T-cells and CD4 T-regulatory cells, but can also be moderately expressed in B-cells and myeloid cells (18–20). DecontPro reduced the PD-1 expression significantly in the naïve T-cells population, while preserving the expression level in the CD4 memory T-cells, CD8 memory T-cells, and CD4 Treg clusters where a high level of PD-1 was expected (Figure 5D). Additionally, some levels in the B-cells and monocytes clusters were also retained. Dsb and totalVI largely removed PD-1 expression in all clusters except CD4 memory T-cells, CD8 memory T-cells, and CD4 Treg cluster, whereas scAR reduced its expression in the CD4 Treg cluster.

CITE-seq panels with larger numbers of antibodies are being rapidly adopted. We also examined DecontPro performance on a larger dataset which profiled cells from lung tumors with a panel of 81 antibodies (21). Using the characteristic markers T cell: CD3; B cell: CD19; Natural Killer cell: CD56; plasmacytoid dendritic cells: CD123, markers are generally only preserved in native cell type clusters (Supplementary Figure S18A). Although the T-cell marker CD3 was still detected in non-native populations such as B-cells and pDCs, the expression of CD3 was largely reduced in all populations except for T cells (Supplementary Figure S18B).

## Discussion

We developed DecontPro, a Bayesian statistical model, that decontaminates two sources of contamination that were observed empirically in CITE-seq data. Previously, the empty drops were thought to only contain noise from ambient material from the cell suspension. Our analyses suggest that the ‘empty droplets’ identified only using RNA are actually heterogeneous in the corresponding ADT data and may contain different sources of signal and noise. One cluster of empty droplets had high levels of specific ADTs and thus likely contained true cells. These results suggest the need for empty drop calling to be performed separately for ADT and RNA data. We also identified a cluster of empty droplets with medium levels of ADTs containing a different profile from that of the ambient droplets. We named these droplets ‘spongelets’ because they had medium levels for most ADTs without any enrichment for specific ADTs compared to true cell clusters. Although our analysis did not reveal the source of the spongelets, we hypothesize that they could be due to the presence of debris from the dissociation procedure or dying cells with a permeable membrane. When generating CITE-seq data, the workflow for dissociation of single cells into suspension and the staining of cells using antibodies is similar to the workflow used in flow cytometry experiments. In flow cytometry, cell fragments and debris are routinely excluded with specific gating strategies (22), whereas such gatings are not performed in CITE-seq as the droplets are typically excluded during empty droplets calling. One study suggests that commonly recommended concentrations for antibodies cocktails in CITE-seq protocols are prone to over-saturation (23). This phenomenon may partially explain the abundance of ADTs we observe in the spongelets. Further work will be required to identify the source of this artifact and understand how experimental parameters can be modified to decrease the contribution of spongelets to the background noise in droplets containing true cells.

These observations motivated us to design a decontamination algorithm that decomposes the ADT count matrix into three components: (i) native counts representing the contribution from true cells, (ii) ambient counts representing the contribution from ambient material and (iii) background counts representing the contribution from other low-level contamination sources such as non-specific binding or spongelets. Having separate estimates of ambient and background contamination can be useful in quality control and testing of experimental parameters. For example, the amount of ambient material may be affected by the type of sorting and enrichment procedures while the amount of background noise may be influenced by the length of antibody conjugation. Importantly, this model allows DecontPro to work in situations where the raw empty droplet matrix is not available which is often the case in public repositories. To estimate the ambient profile of a dataset when the empty drop matrix is not available, we use the average of the true cells. We found that the average of the true cells was highly correlated to the ambient droplets in three out of the four datasets examined. The lower correlation in the Golomb dataset was primarily due to one ADT (Ly6C) which had lower than expected levels in the cells given the level in the ambient droplets. Other experimental procedures such as cell sorting for particular populations may also break the assumption that the cell average will accurately approximate the profile of the ambient droplets. In these cases, our software allows users to input the empty droplets to more accurately estimate the ambient profile.

Overall, DecontPro can be used as an important quality assessment tool that estimates the levels of different sources contributing to the contamination in ADT data. The computational decontamination of ADT counts with DecontPro will aid in downstream clustering and visualization and can be systematically included in analysis workflows.

## Supplementary Material

gkad1032_Supplemental_File

## Data Availability

Data for PBMC 10k obtained by 10x Genomics were downloaded from 10x Genomics website (https://www.10xgenomics.com/resources/datasets/10-k-pbm-cs-from-a-healthy-donor-gene-expression-and-cell-surface-protein-3-standard-3-0-0). Data for PBMC 5k obtained by 10x Genomics were downloaded from 10x Genomics website (https://www.10xgenomics.com/resources/datasets/5-k-peripheral-blood-mononuclear-cells-pbm-cs-from-a-healthy-donor-with-cell-surface-proteins-next-gem-3-1-standard-3-1-0). Data for MALT 10k obtained by 10x Genomics were downloaded from 10x Genomics website (https://www.10xgenomics.com/resources/datasets/10-k-cells-from-a-malt-tumor-gene-expression-and-cell-surface-protein-3-standard-3-0-0). Golomb dataset were downloaded from GEO with accession number GSE148127 and obtained from the corresponding author of the study. DecontPro is freely available as an R package on GitHub: https://github.com/campbio/decontX. The code underlying this article is available in Zenodo at http://doi.org/10.5281/zenodo.10005307.

## References

[1] Stoeckius M. , Hafemeister C., Stephenson W., Houck-Loomis B., Chattopadhyay P.K., Swerdlow H., Satija R., Smibert P. Simultaneous epitope and transcriptome measurement in single cells. Nat. Methods. 2017; 14:865–868. 28759029 10.1038/nmeth.4380 PMC5669064

[2] Nathan A. , Beynor J.I., Baglaenko Y., Suliman S., Ishigaki K., Asgari S., Huang C.-C., Luo Y., Zhang Z., Lopez K. et al. Multimodally profiling memory T cells from a tuberculosis cohort identifies cell state associations with demographics, environment and disease. Nat. Immunol. 2021; 22:781–793. 34031617 10.1038/s41590-021-00933-1 PMC8162307

[3] Cadot S. , Valle C., Tosolini M., Pont F., Largeaud L., Laurent C., Fournie J.J., Ysebaert L., Quillet-Mary A. Longitudinal CITE-Seq profiling of chronic lymphocytic leukemia during ibrutinib treatment: evolution of leukemic and immune cells at relapse. Biomark. Res. 2020; 8:72. 33298182 10.1186/s40364-020-00253-w PMC7724843

[4] Wu S.Z. , Al-Eryani G., Roden D.L., Junankar S., Harvey K., Andersson A., Thennavan A., Wang C., Torpy J.R., Bartonicek N. et al. A single-cell and spatially resolved atlas of human breast cancers. Nat. Genet. 2021; 53:1334–1347. 34493872 10.1038/s41588-021-00911-1 PMC9044823

[5] Ben-Chetrit N. , Niu X., Swett A.D., Sotelo J., Jiao M.S., Stewart C.M., Potenski C., Mielinis P., Roelli P., Stoeckius M. et al. Integration of whole transcriptome spatial profiling with protein markers. Nat. Biotechnol. 2023; 41:788–793. 36593397 10.1038/s41587-022-01536-3 PMC10272089

[6] Mimitou E.P. , Cheng A., Montalbano A., Hao S., Stoeckius M., Legut M., Roush T., Herrera A., Papalexi E., Ouyang Z. et al. Multiplexed detection of proteins, transcriptomes, clonotypes and CRISPR perturbations in single cells. Nat. Methods. 2019; 16:409–412. 31011186 10.1038/s41592-019-0392-0 PMC6557128

[7] Mimitou E.P. , Lareau C.A., Chen K.Y., Zorzetto-Fernandes A., Hao Y., Takeshima Y., Luo W., Huang T.-S., Yeung B.Z., Papalexi E. et al. Scalable, multimodal profiling of chromatin accessibility, gene expression and protein levels in single cells. Nat. Biotechnol. 2021; 39:1246–1258. 34083792 10.1038/s41587-021-00927-2 PMC8763625

[8] Zheng Y. , Jun S.-H., Tian Y., Florian M., Gottardo R. Robust normalization and integration of single-cell protein expression across CITE-seq datasets. 2022; bioRxiv doi:01 May 2022, preprint: not peer reviewed 10.1101/2022.04.29.489989.

[9] Gayoso A. , Steier Z., Lopez R., Regier J., Nazor K.L., Streets A., Yosef N. Joint probabilistic modeling of single-cell multi-omic data with totalVI. Nat. Methods. 2021; 18:272–282. 33589839 10.1038/s41592-020-01050-x PMC7954949

[10] Young M.D. , Behjati S. SoupX removes ambient RNA contamination from droplet-based single-cell RNA sequencing data. Gigascience. 2020; 9:giaa151. 33367645 10.1093/gigascience/giaa151 PMC7763177

[11] Yang S. , Corbett S.E., Koga Y., Wang Z., Johnson W.E., Yajima M., Campbell J.D. Decontamination of ambient RNA in single-cell RNA-seq with DecontX. Genome Biol. 2020; 21:57. 32138770 10.1186/s13059-020-1950-6 PMC7059395

[12] Mulè M.P. , Martins A.J., Tsang J.S. Normalizing and denoising protein expression data from droplet-based single cell profiling. Nat. Commun. 2022; 13:2099. 35440536 10.1038/s41467-022-29356-8 PMC9018908

[13] Sheng C. , Lopes R., Li G., Schuierer S., Waldt A., Cuttat R., Dimitrieva S., Kauffmann A., Durand E., Galli G.G. et al. Probabilistic machine learning ensures accurate ambient denoising in droplet-based single-cell omics. 2022; bioRxiv doi:24 March 2022, preprint: not peer reviewed 10.1101/2022.01.14.476312.

[14] Golomb S.M. , Guldner I.H., Zhao A., Wang Q., Palakurthi B., Aleksandrovic E.A., Lopez J.A., Lee S.W., Yang K., Zhang S. Multi-modal single-cell analysis reveals brain immune landscape plasticity during aging and gut microbiota dysbiosis. Cell Rep. 2020; 33:108438. 33264626 10.1016/j.celrep.2020.108438 PMC7737488

[15] Hao Y. , Hao S., Andersen-Nissen E., Mauck W.M., Zheng S., Butler A., Lee M.J., Wilk A.J., Darby C., Zager M. et al. Integrated analysis of multimodal single-cell data. Cell. 2021; 184:3573–3587. 34062119 10.1016/j.cell.2021.04.048 PMC8238499

[16] Grieshaber-Bouyer R. , Radtke F.A., Cunin P., Stifano G., Levescot A., Vijaykumar B., Nelson-Maney N., Blaustein R.B., Monach P.A., Nigrovic P.A. et al. The neutrotime transcriptional signature defines a single continuum of neutrophils across biological compartments. Nat. Commun. 2021; 12:2856. 34001893 10.1038/s41467-021-22973-9 PMC8129206

[17] Kim M. , Lu R.J., Benayoun B.A. Single-cell RNA-seq of primary bone marrow neutrophils from female and male adult mice. Sci. Data. 2022; 9:442. 35871169 10.1038/s41597-022-01544-7 PMC9308797

[18] Jubel J.M. , Barbati Z.R., Burger C., Wirtz D.C., Schildberg F.A. The role of PD-1 in acute and chronic infection. Front. Immunol. 2020; 11:487. 32265932 10.3389/fimmu.2020.00487 PMC7105608

[19] Okazaki T. , Chikuma S., Iwai Y., Fagarasan S., Honjo T. A rheostat for immune responses: the unique properties of PD-1 and their advantages for clinical application. Nat. Immunol. 2013; 14:1212–1218. 24240160 10.1038/ni.2762

[20] Francisco L.M. , Sage P.T., Sharpe A.H. The PD-1 pathway in tolerance and autoimmunity. Immunol. Rev. 2010; 236:219–242. 20636820 10.1111/j.1600-065X.2010.00923.x PMC2919275

[21] Leader A.M. , Grout J.A., Maier B.B., Nabet B.Y., Park M.D., Tabachnikova A., Chang C., Walker L., Lansky A., Le Berichel J. et al. Single-cell analysis of human non-small cell lung cancer lesions refines tumor classification and patient stratification. Cancer Cell. 2021; 39:1594–1609. 34767762 10.1016/j.ccell.2021.10.009 PMC8728963

[22] Cossarizza A. , Chang H.-D., Radbruch A., Abrignani S., Addo R., Akdis M., Andrä I., Andreata F., Annunziato F., Arranz E. et al. Guidelines for the use of flow cytometry and cell sorting in immunological studies (third edition). Eur. J. Immunol. 2021; 51:2708–3145. 34910301 10.1002/eji.202170126 PMC11115438

[23] Buus T.B. , Herrera A., Ivanova E., Mimitou E., Cheng A., Herati R.S., Papagiannakopoulos T., Smibert P., Odum N., Koralov S.B. Improving oligo-conjugated antibody signal in multimodal single-cell analysis. eLife. 2021; 10:e61973. 33861199 10.7554/eLife.61973 PMC8051954

