# Supplementary material for: Characterization and decontamination of background noise in droplet-based single-cell protein expression data with DecontPro
Source: Nucleic Acids Res. 2023 Nov 17;52(1):e4. doi: 10.1093/nar/gkad1032 (PMC10783508; doi:10.1093/nar/gkad1032)
Supplement: gkad1032_Supplemental_File [file gkad1032_supplemental_file.pdf]

Table S1. Markers used to calculate positive and negative scores.

Dataset	Cell Cluster	Scores	Marker
**PBMC 10K**	CD14 Monocyte	+	CD14
	CD4 naïve T-cell	+	CD4, CD45RA
		-	CD8a, CD45RO
	CD4 memory T-cell	+	CD4, CD45RO
		-	CD8a, CD45RA
	NK	+	CD16, CD56
	CD8 memory T-cell	+	CD8a, CD45RO
		-	CD4, CD45RA
	B	+	CD19
		-	CD3
	CD8 naïve T-cell	+	CD8a, CD45RA
		-	CD4, CD45RO
	CD16 monocyte	+	CD16
**PBMC 5K**	CD14 Monocyte	+	CD14
	CD4 naïve T-cell	+	CD4, CD45RA
		-	CD8a, CD45RO
	CD4 memory T-cell	+	CD4, CD45RO
		-	CD8a, CD45RA
	NK	+	CD16, CD56
	CD8 memory T-cell	+	CD8a, CD45RO
		-	CD4, CD45RA
	B	+	CD19
		-	CD3
**MALT 10K**	CD4 memory T-cell	+	CD4, CD45RO
		-	CD8a, CD45RA
	CD8 memory T-cell	+	CD8a, CD45RO
		-	CD4, CD45RA
	B	+	CD19
		-	CD3
**Golomb**	Microglia	+	CD11b
		-	CD3, CD8a
	CD8 T-cell	+	CD3, CD8a
		-	CD45R-B220
	B	+	CD45R-B220
		-	CD3, CD8a

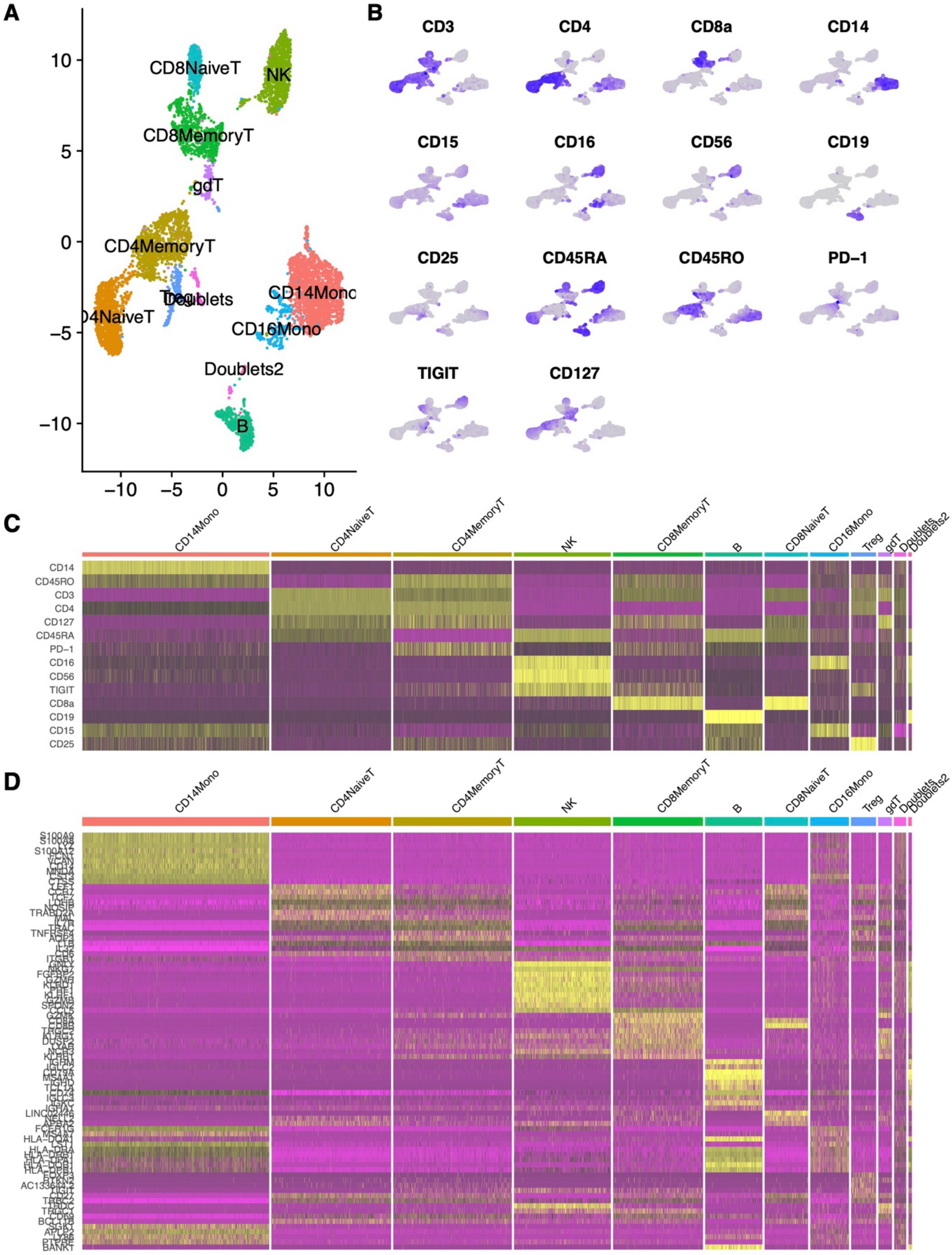

Figure S1. Annotating PBMC 10K dataset. A. B. UMAP and feature plots of ADTs. **C.** Differential expression analysis for each cluster using the ADT expression data. **D.** Differential expression analysis for each cluster using the gene expression data.

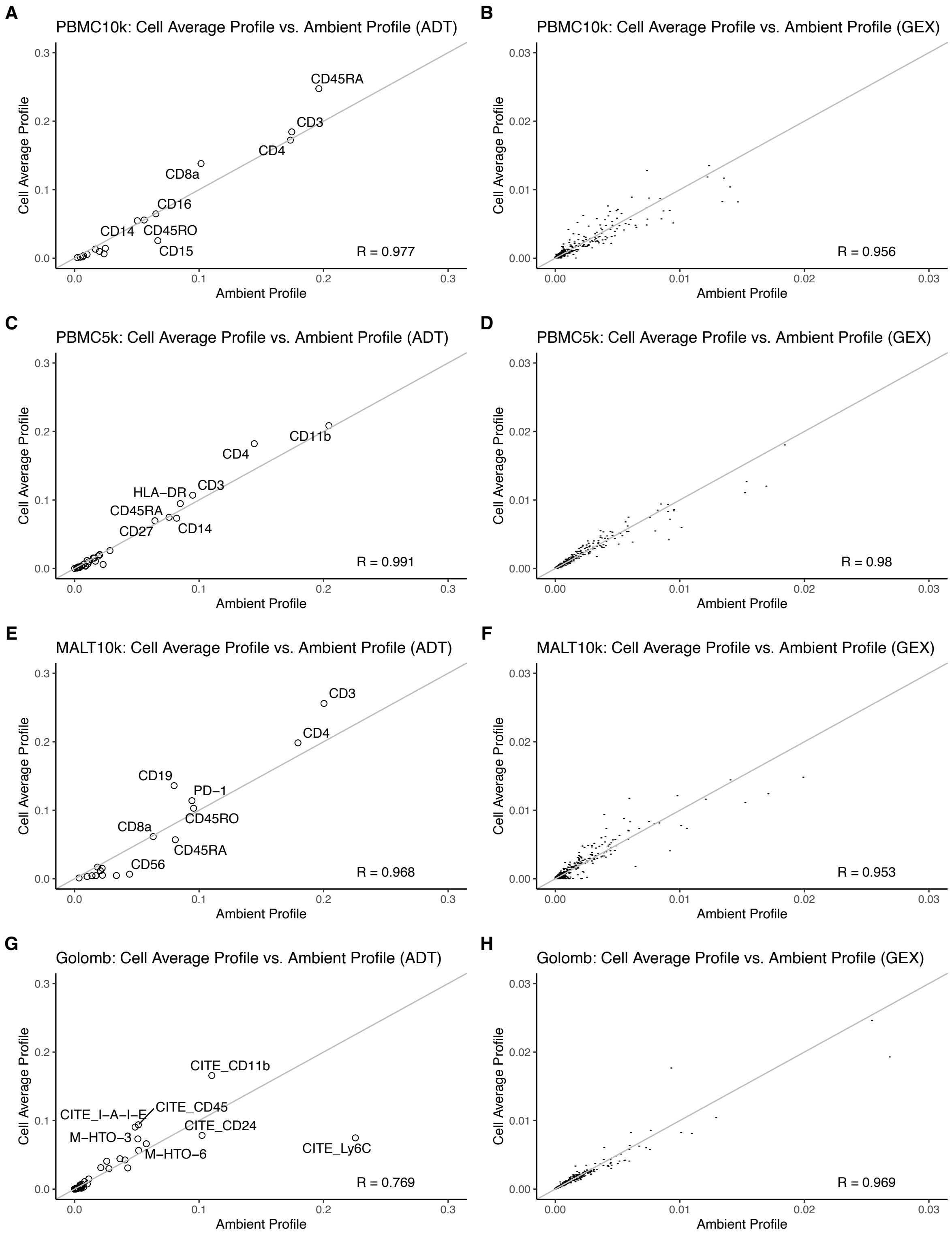

Figure S2. Correlation between Cell profile and Ambient profile. The average cell profile of ADTs (**A. C. E. G.**) from the Cell droplets was found to be highly correlated with the ambient profile which represents the ambient signal. This finding was reproduced using the gene expression data of the droplets (**B. D. F. H.**).

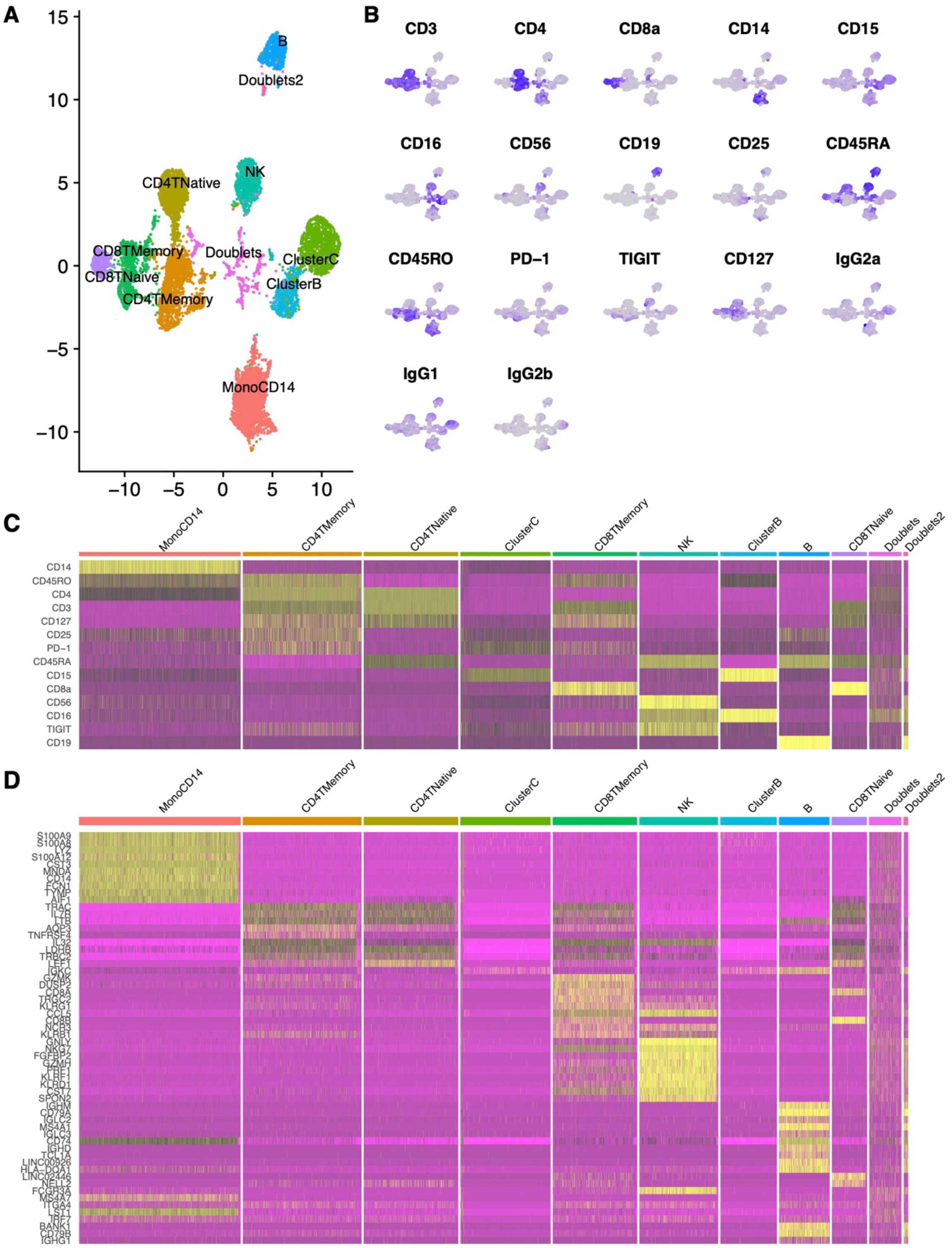

Figure S3. Annotating PBMC 10K dataset with Cluster B and Cluster C. A. UMAP of droplets from the Cell, Cluster B and Cluster C. One thousand droplets were each randomly sampled from the Cluster B and C to join the Cell cluster for clustering. **B.** Feature plot of ADTs on the UMAP. **C.** Differential expression analysis for each cluster using the ADT expression data. **D.** Differential expression analysis for each cluster using the gene expression data.

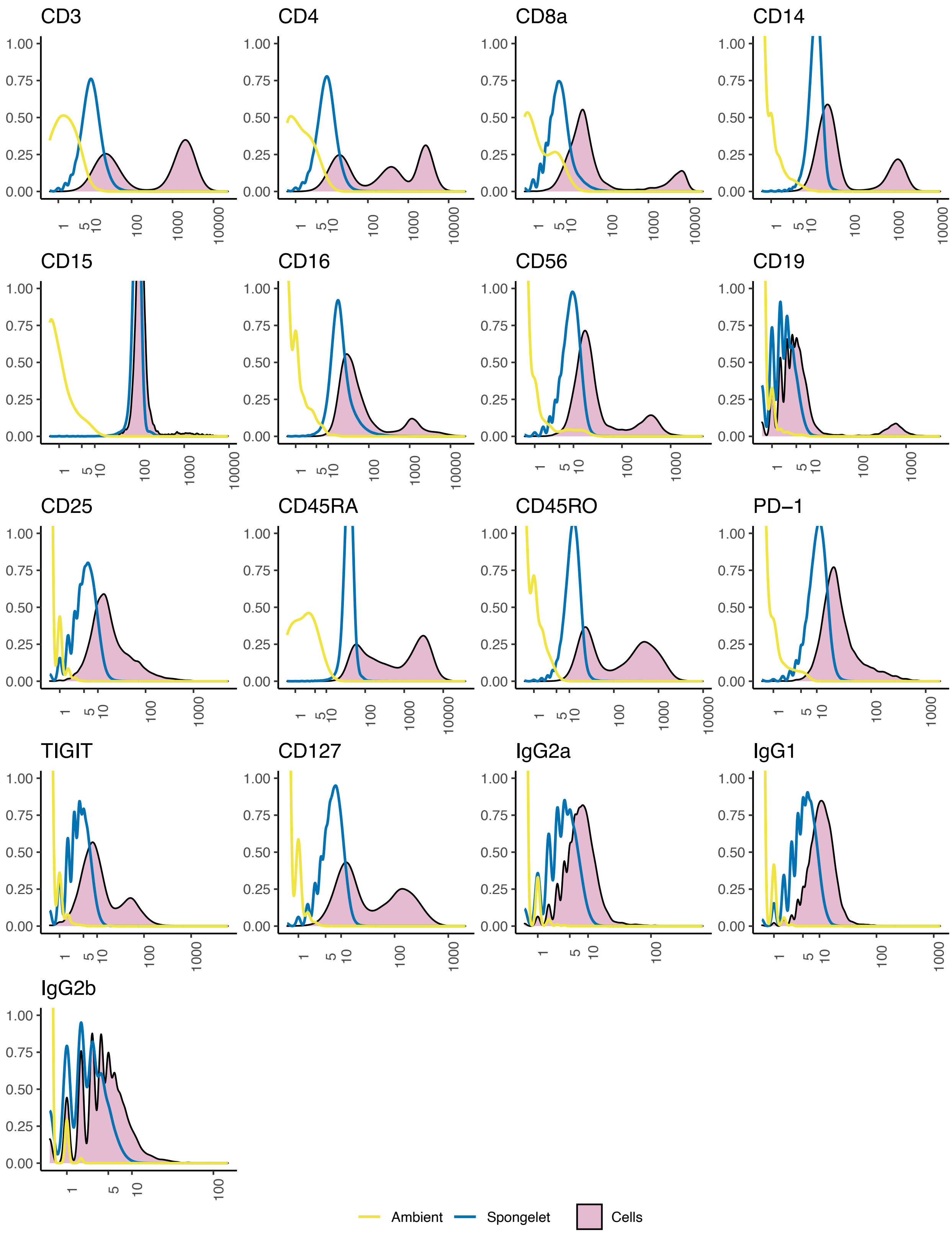

Figure S4. Density plot for ADTs in PBMC 10K dataset. Density plots of all ADTs in the dataset for droplets from the Cell, Spongelet and Ambient clusters.

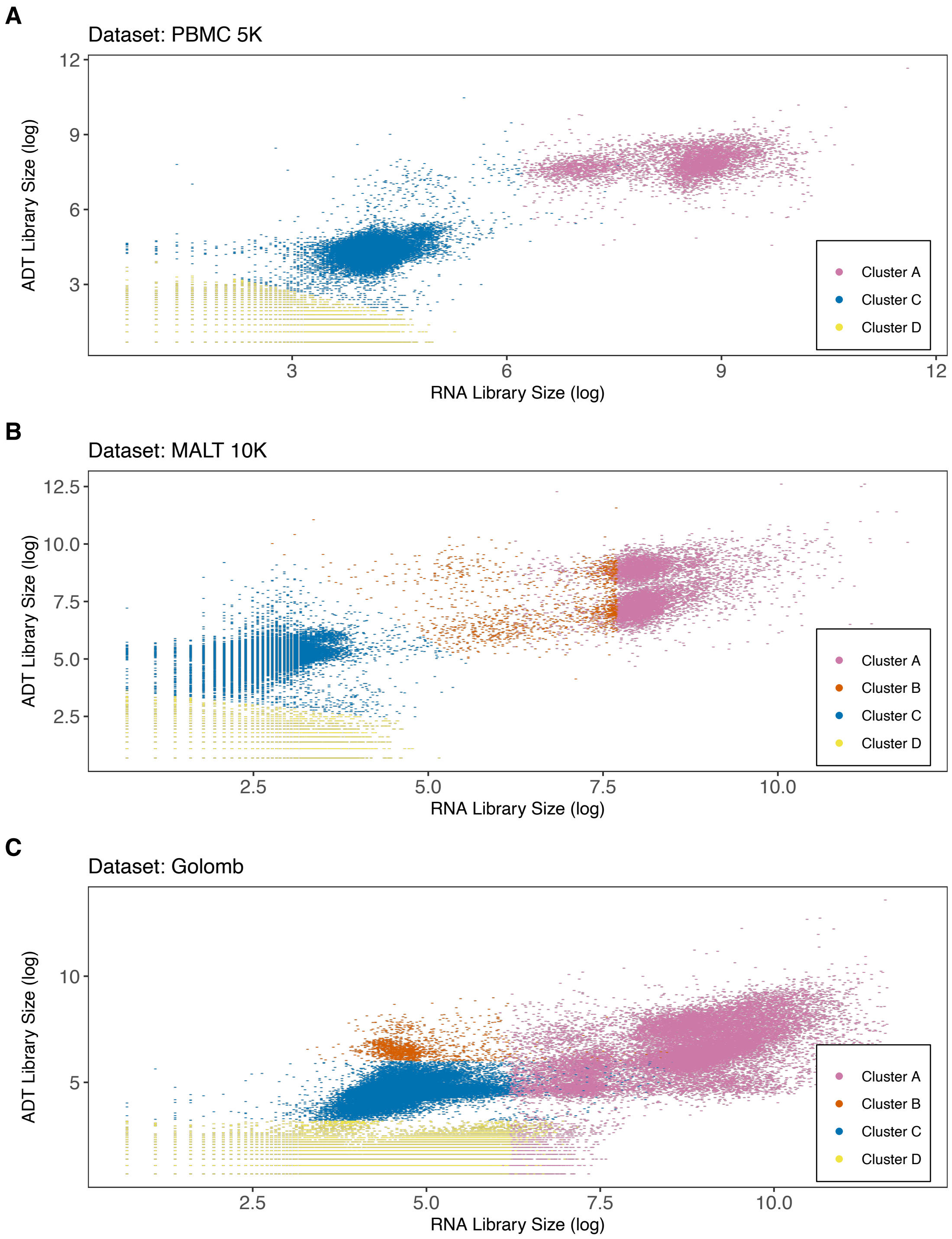

Figure S5. Identifying clusters in empty droplets for three datasets. CellRanger was used to distinguish empty droplets from true cells. The true cells are labeled as “Cluster A” while the rest of the empty droplets were clustered with k-means. The only exception is the Golomb dataset where clusters among the empty drops were manually identified based on thresholds for ADT and RNA library sizes. Cluster B likely contained true cells with high ADT and low RNA counts. We called the droplets in Cluster C “spongelets” as they had intermediate levels of ADTs along with low RNA content. Droplets in Cluster D contained low levels of both ADT and RNA and matched ambient profiles.

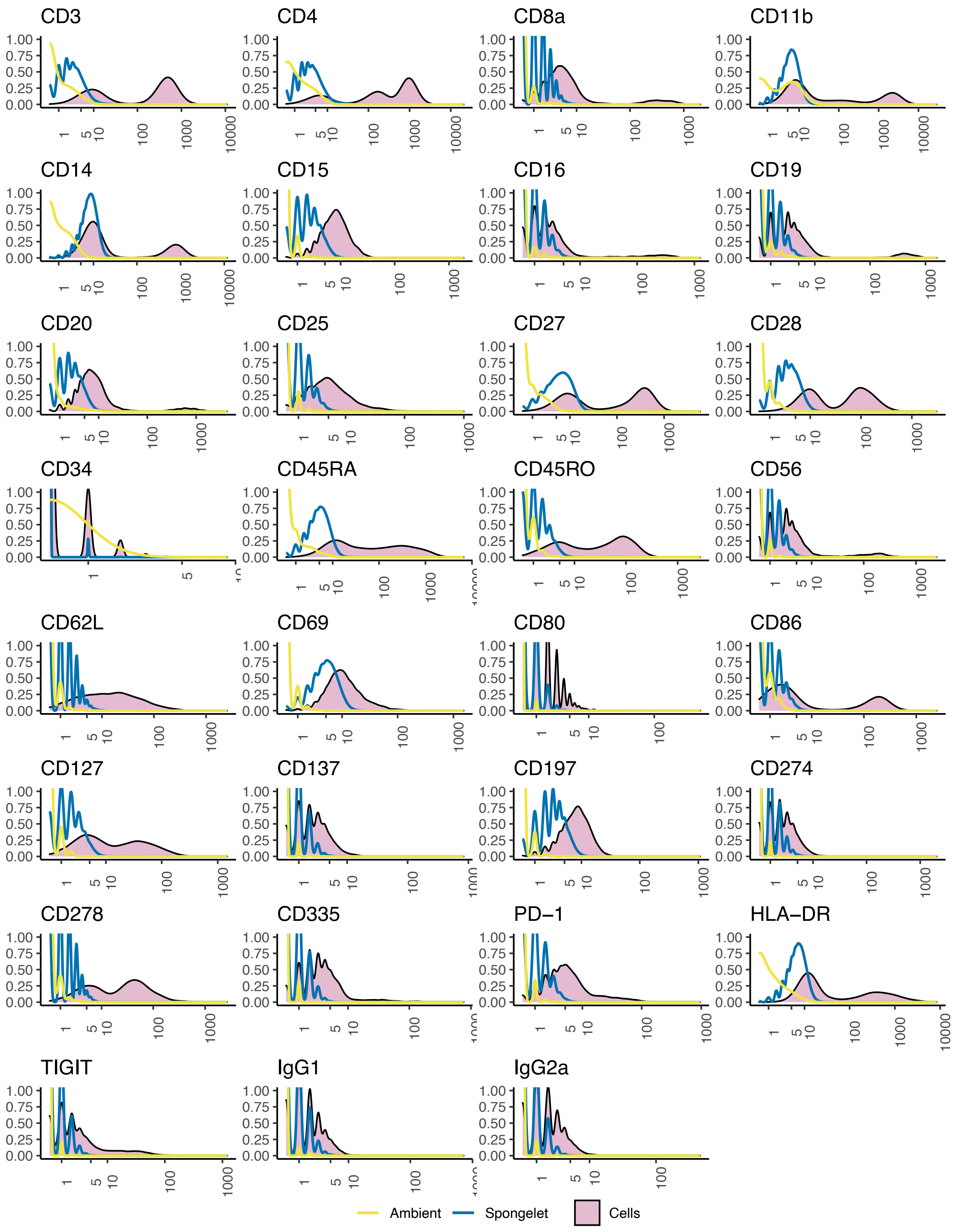

Figure S6. Density plot for ADTs in PBMC 5K dataset. Density plots of all ADTs in the dataset for droplets from the Cell, Spongelet and Ambient clusters.

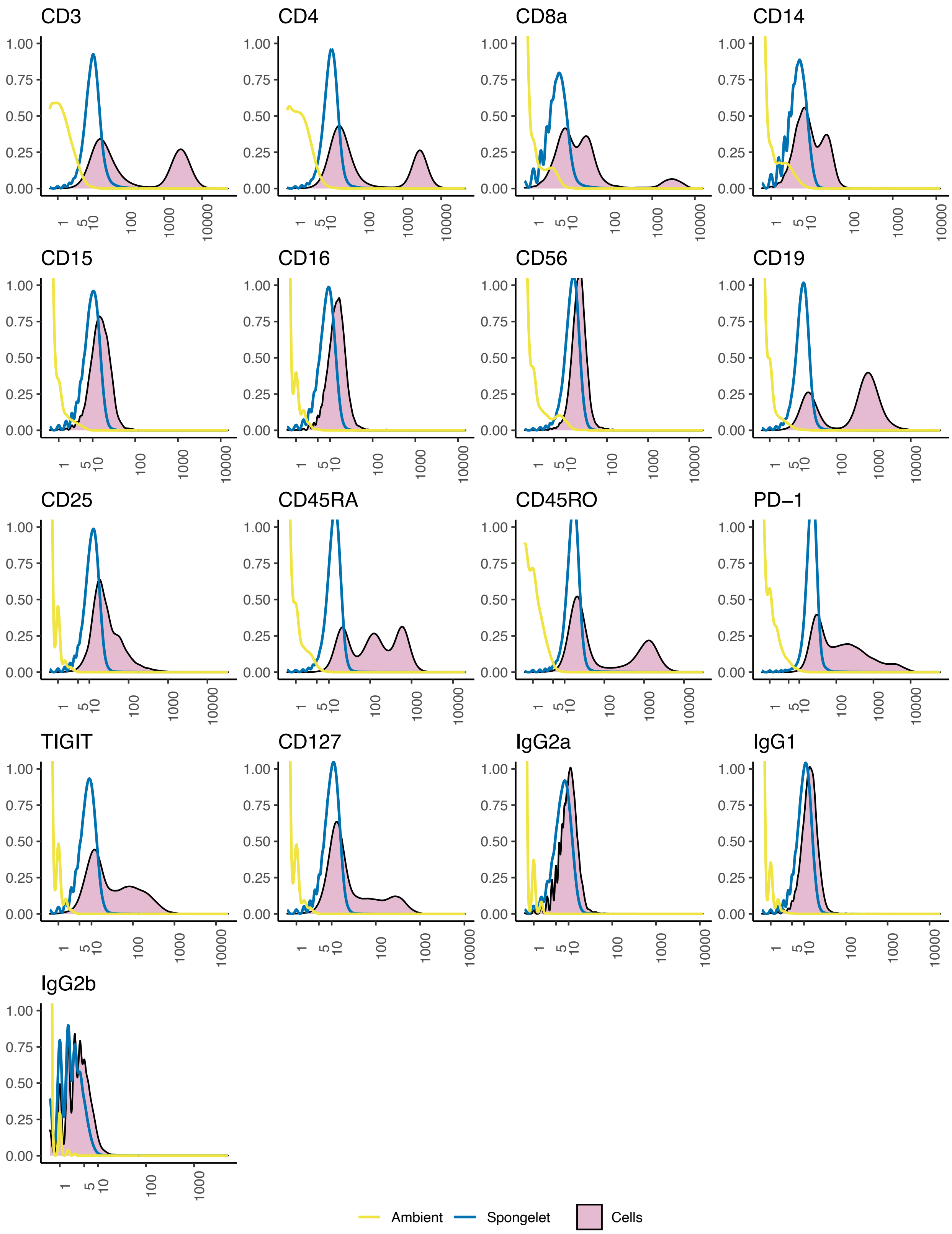

Figure S7. Density plot for ADTs in MALT 10K dataset. Density plots of all ADTs in the dataset for droplets from the Cell, Spongelet and Ambient clusters.

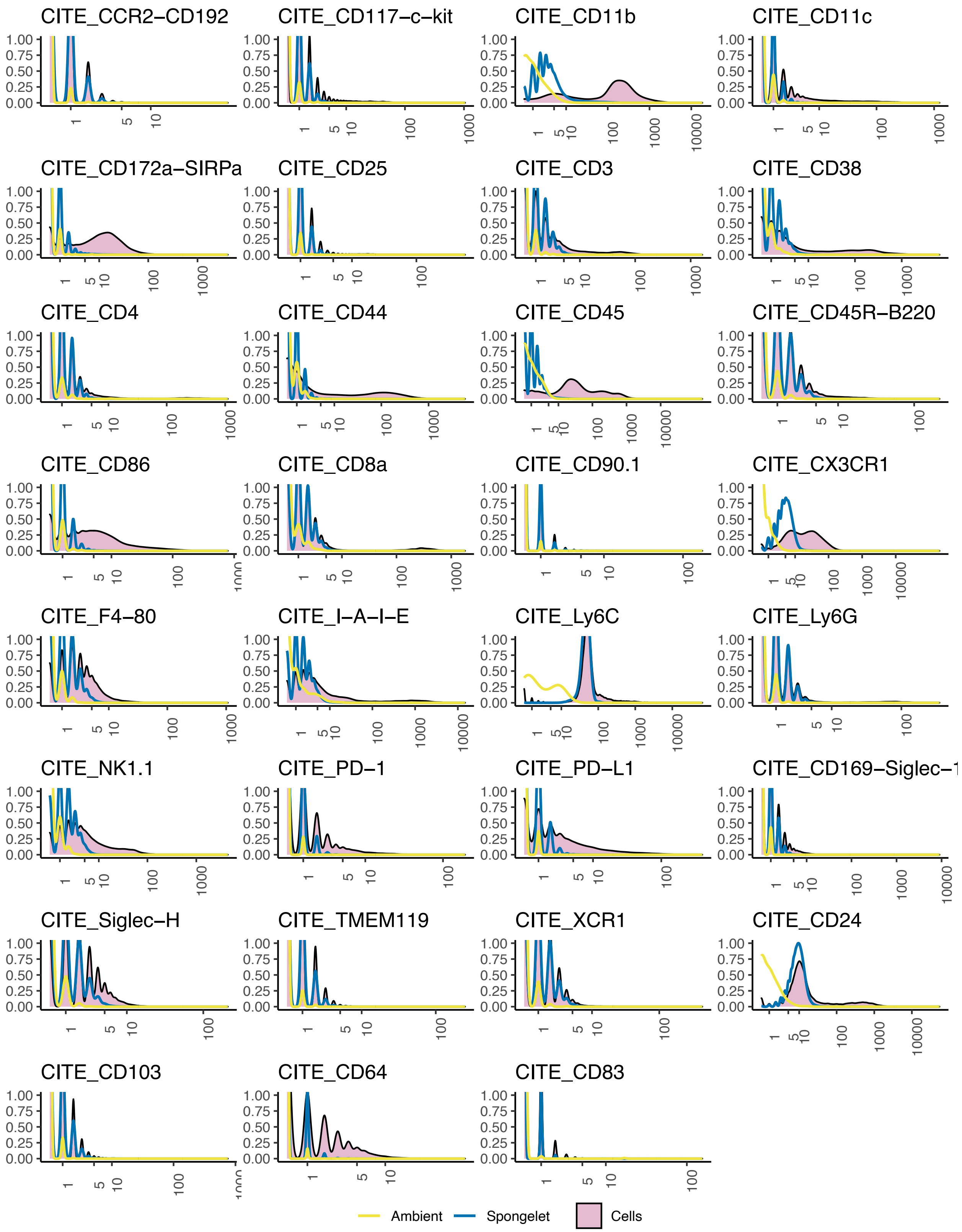

Figure S8. Density plot for ADTs in Golomb dataset. Density plots of all ADTs in the dataset for droplets from the Cell, Spongelet and Ambient clusters.

A

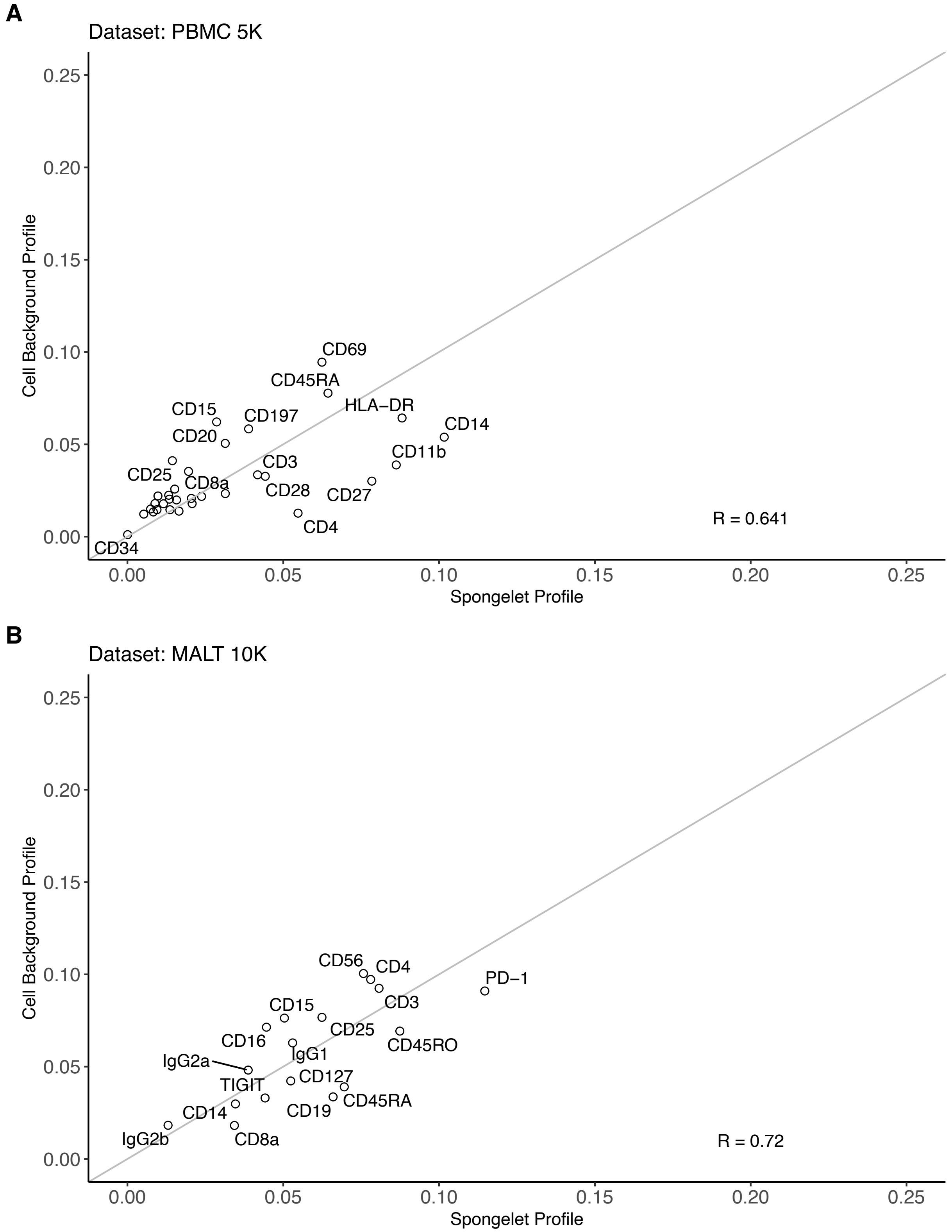

Figure S9. Correlation between Cell background profile and Spongelet profile for **A.** PBMC 5K dataset and **B.** MALT 10K dataset.

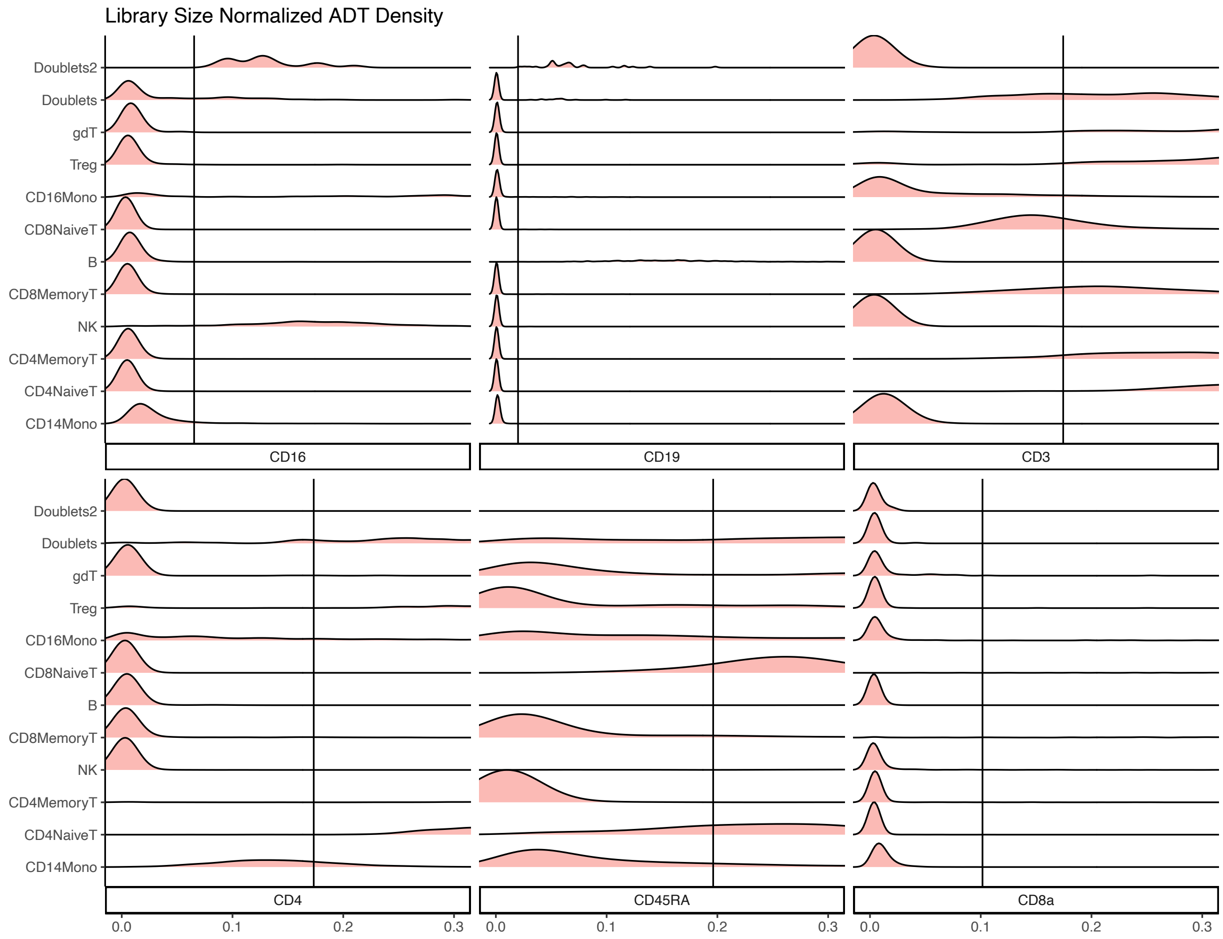

Figure S10. Density plots for library size-normalized droplets for PBMC 10K dataset. ADTs not expected to be expressed in cell types show a peak at a near zero level, in contrast to a more spread-out peak at a higher level when they are natively expressed in other cell types. When overlapping the normalized ambient profile on top of the density plot, the ambient distribution differs from the mean of the margin peaks.

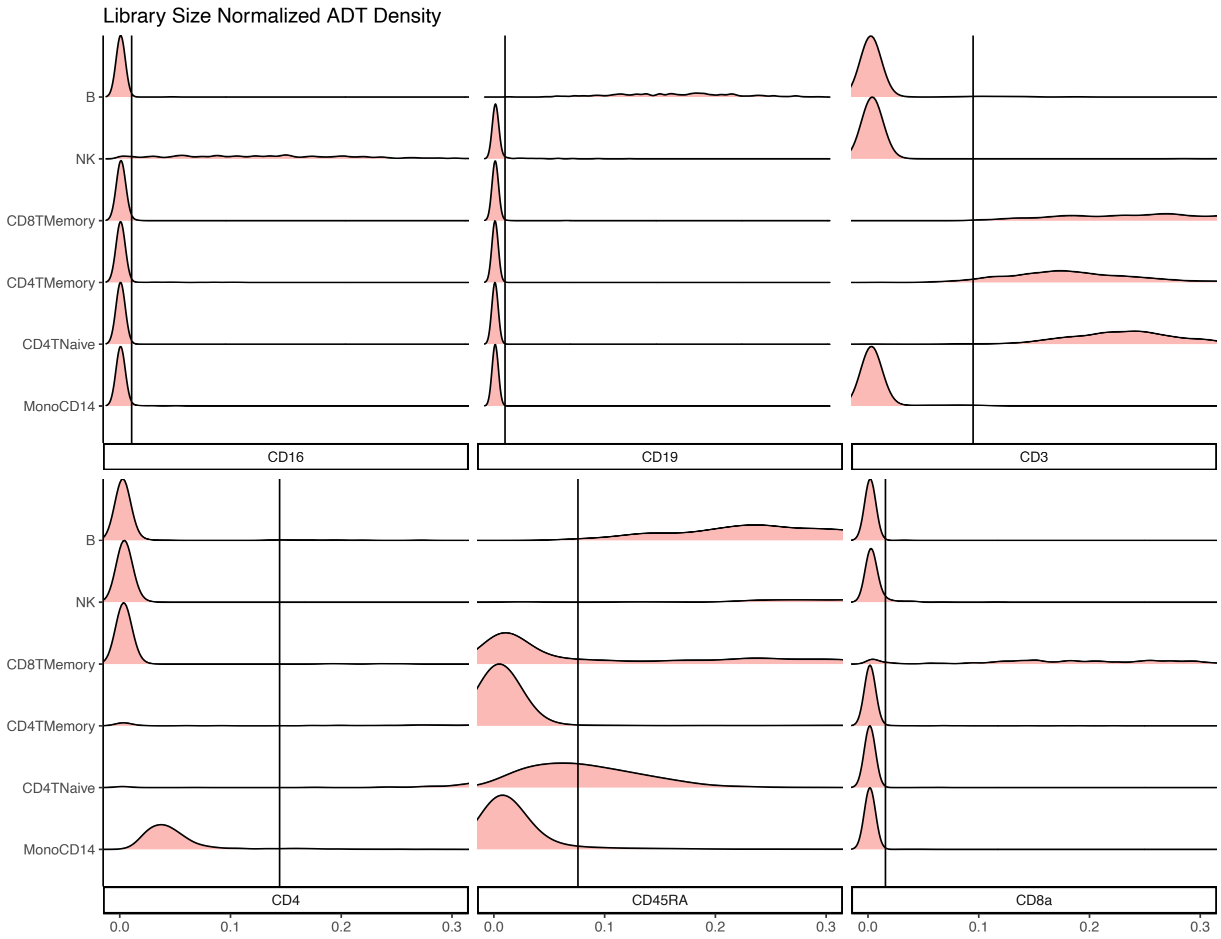

Figure S11. Density plots for library size-normalized droplets for PBMC 5K dataset. ADTs not expected to be expressed in cell types show a peak at a near zero level, in contrast to a more spread-out peak at a higher level when they are natively expressed in other cell types. When overlapping the normalized ambient profile on top of the density plot, the ambient distribution differs from the mean of the margin peaks.

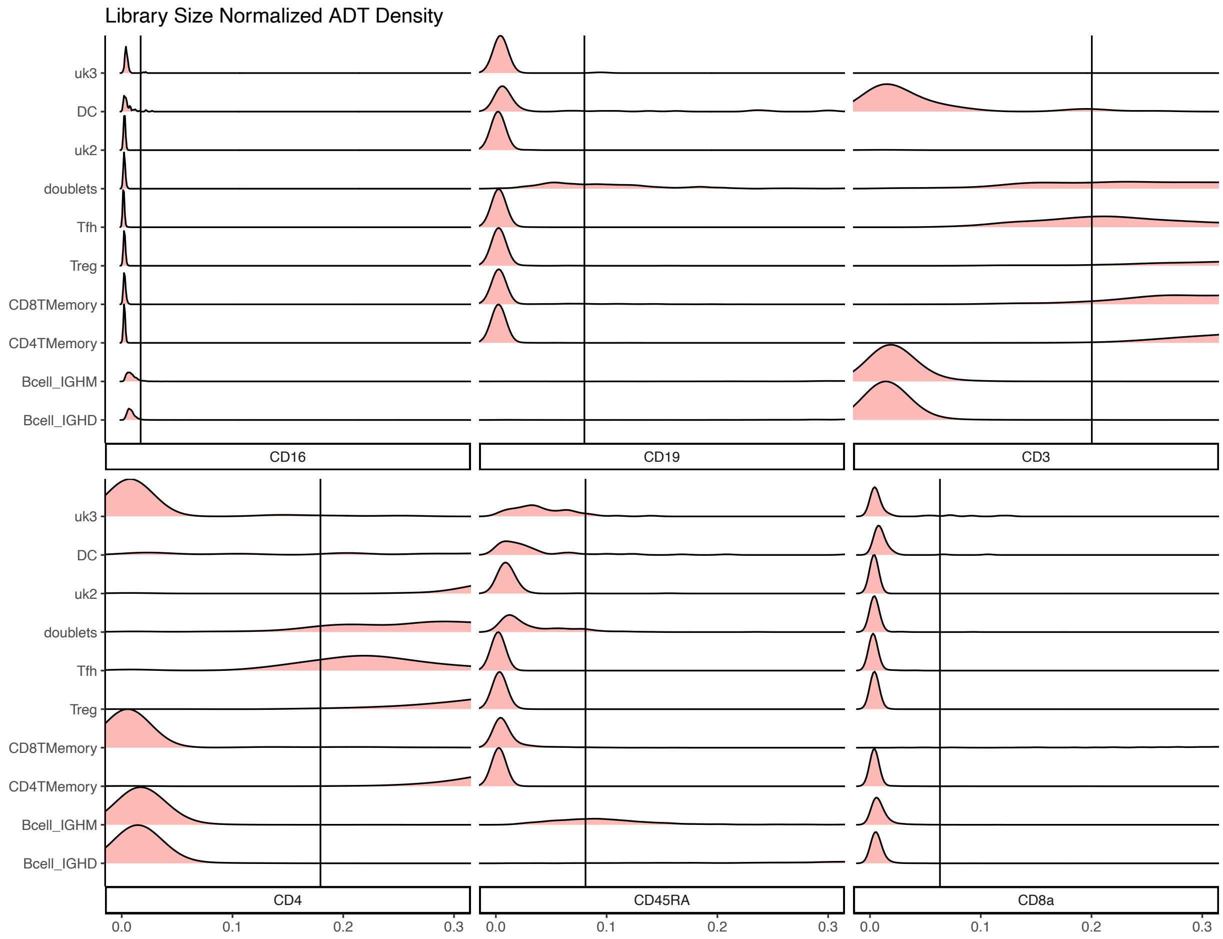

Figure S12. Density plots for library size-normalized droplets for MALT 10K dataset. ADTs not expected to be expressed in cell types show a peak at a near zero level, in contrast to a more spread-out peak at a higher level when they are natively expressed in other cell types. When overlapping the normalized ambient profile on top of the density plot, the ambient distribution differs from the mean of the margin peaks.

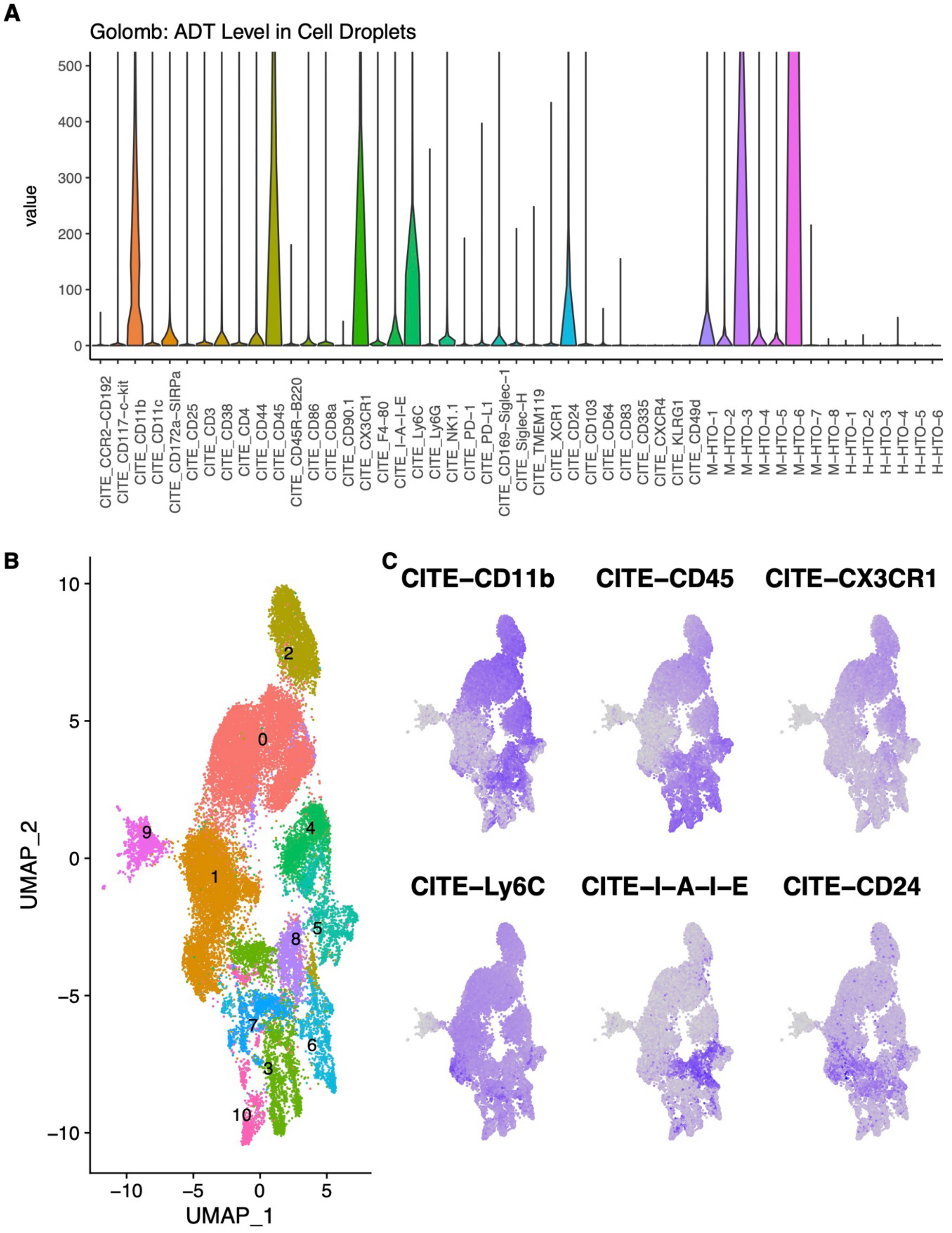

Figure S13. Ly6C as an outlier ADT among the highly stained ADTs in Golomb dataset. A. Violin plot of ADT expression level among the cell droplets. Ly6C is one of the highly stained ADTs in the panel. **B.C.** Highly stained ADTs identified in **A** were shown on a UMAP. Ly6C shows high expression globally, in contrast to other ADTs with localized high expression.

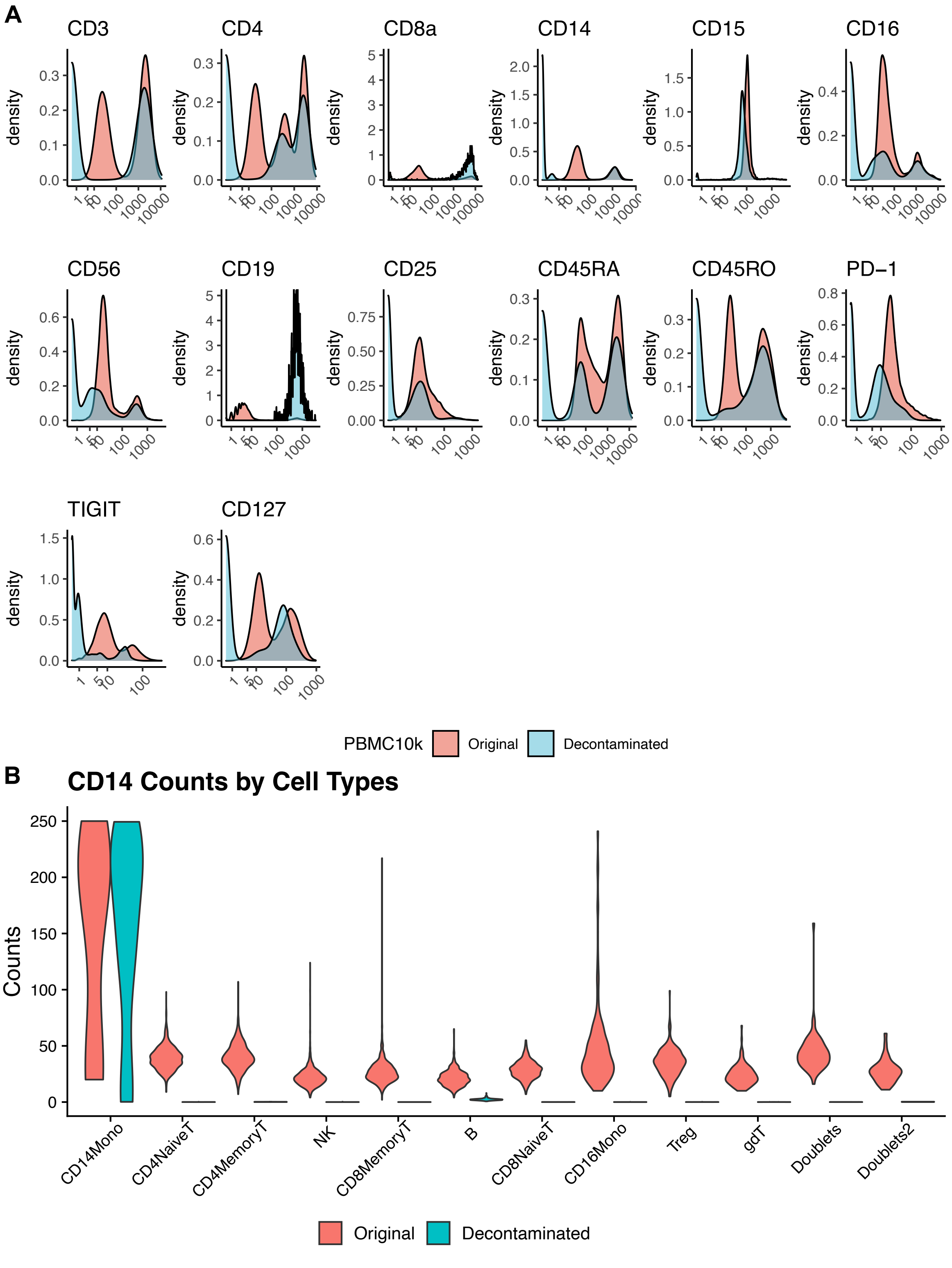

Figure S14. Comparison of uncorrected original counts and decontaminated counts of PBMC 10K dataset after DecontPro. A. Density of each ADT expression before and after decontamination shows the background peak is largely reduced. **B.** CD14 counts are greatly reduced in clusters other than CD14 Monocyte cluster.

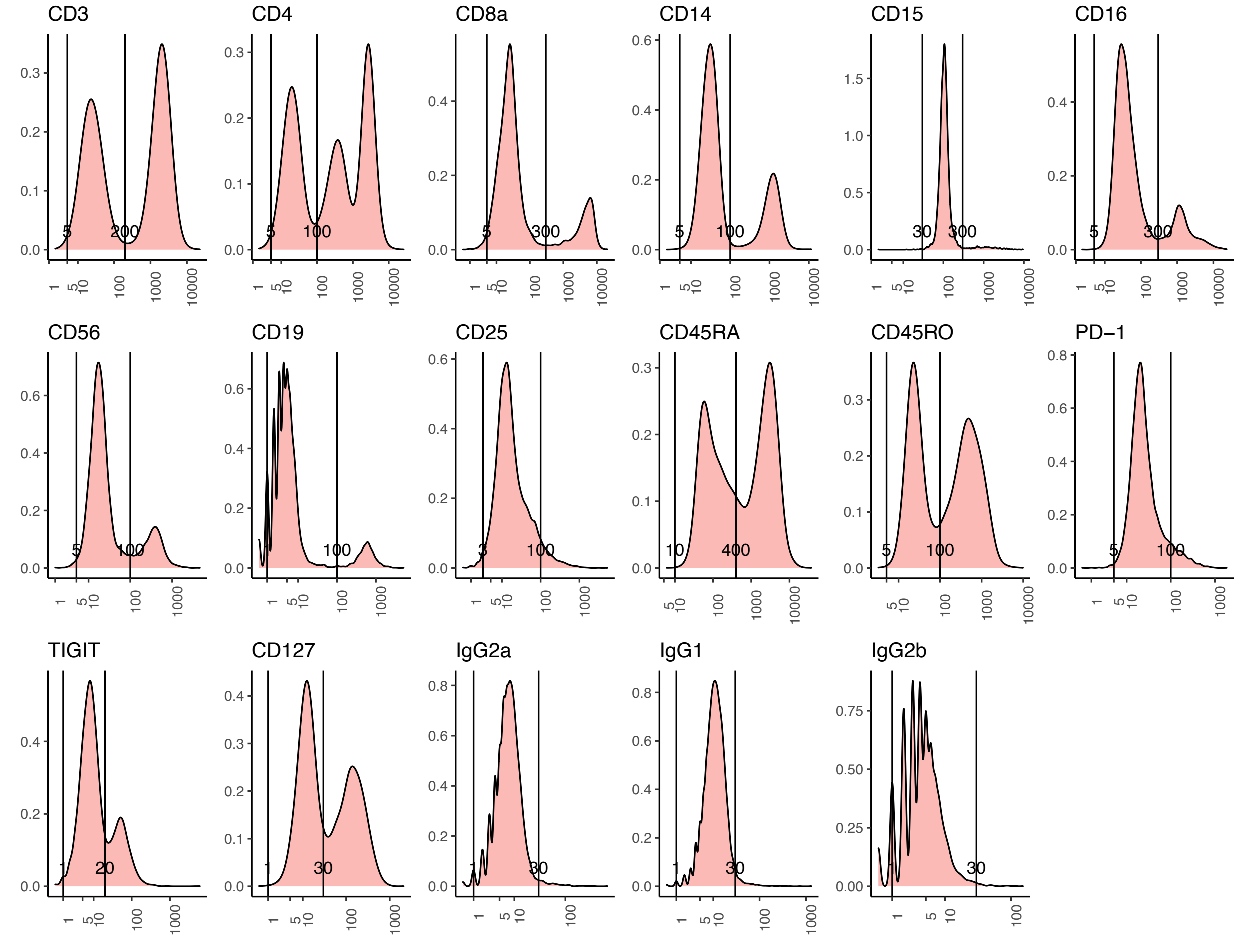

Figure S15. Identifying background peaks for PBMC 10K dataset. Background peaks for ADTs were identified and labeled using an upper bound and a lower bound.

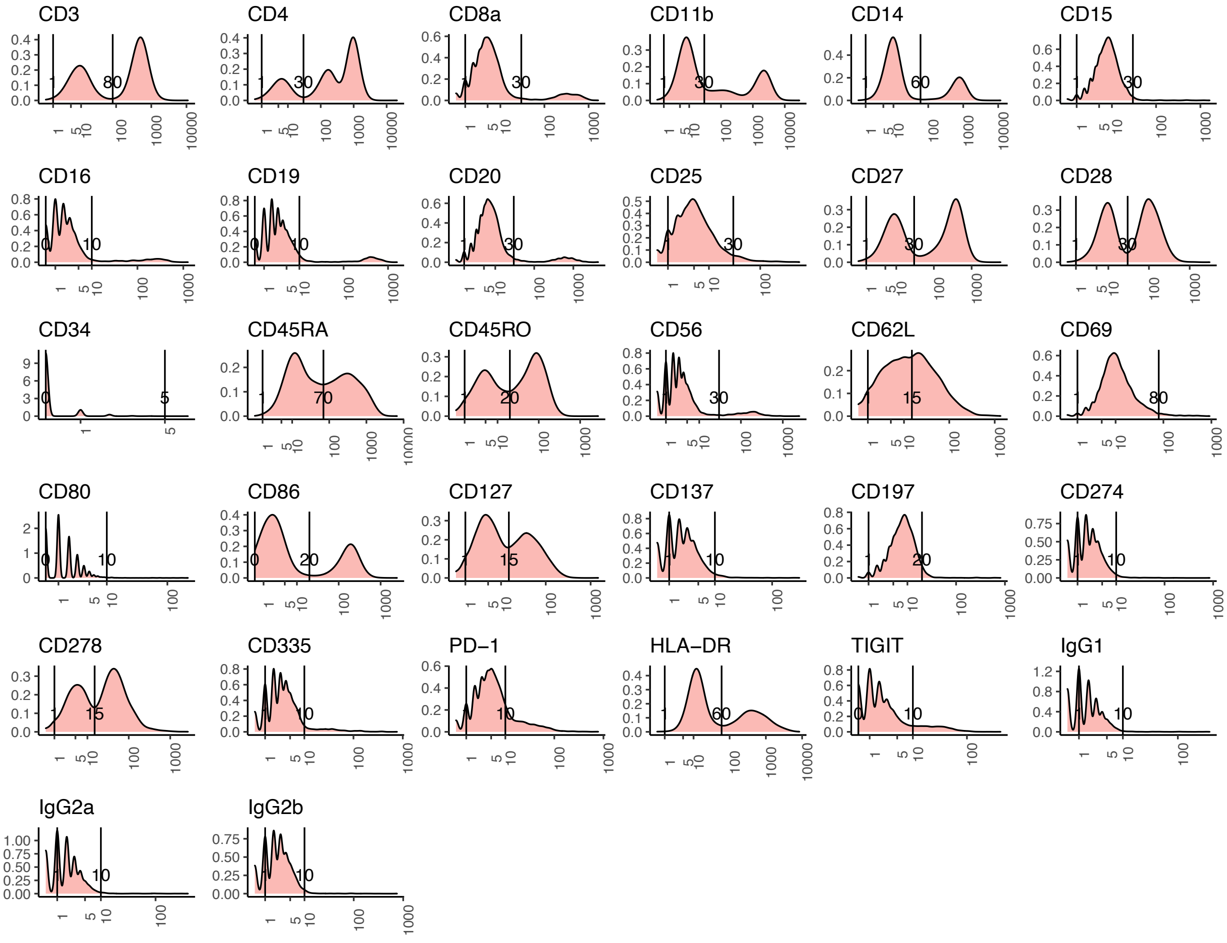

Figure S16. Identifying background peaks for PBMC 5K dataset. Background peaks for ADTs were identified and labeled using an upper bound and a lower bound.

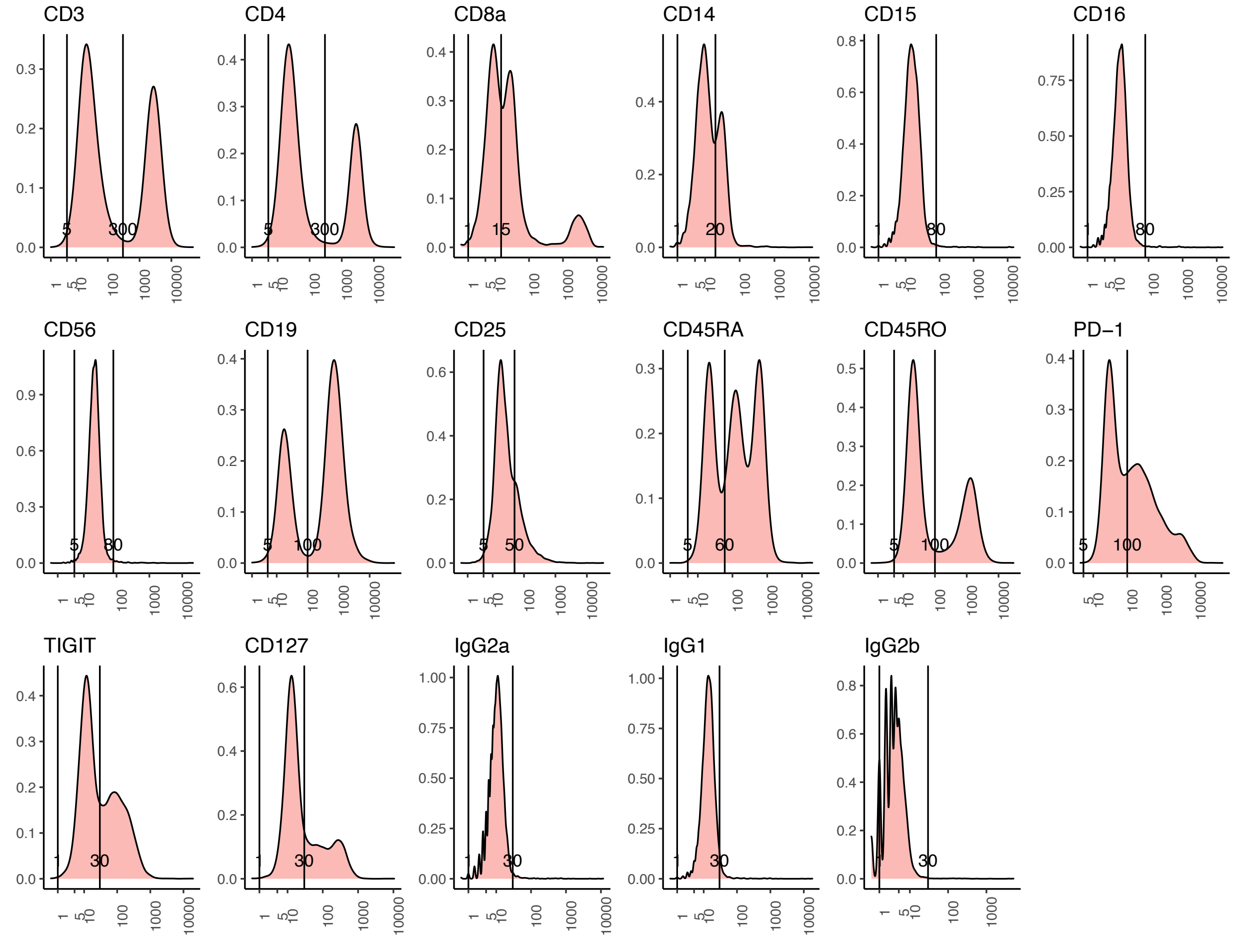

Figure S17. Identifying background peaks for MALT 10K dataset. Background peaks for ADTs were identified and labeled using an upper bound and a lower bound.

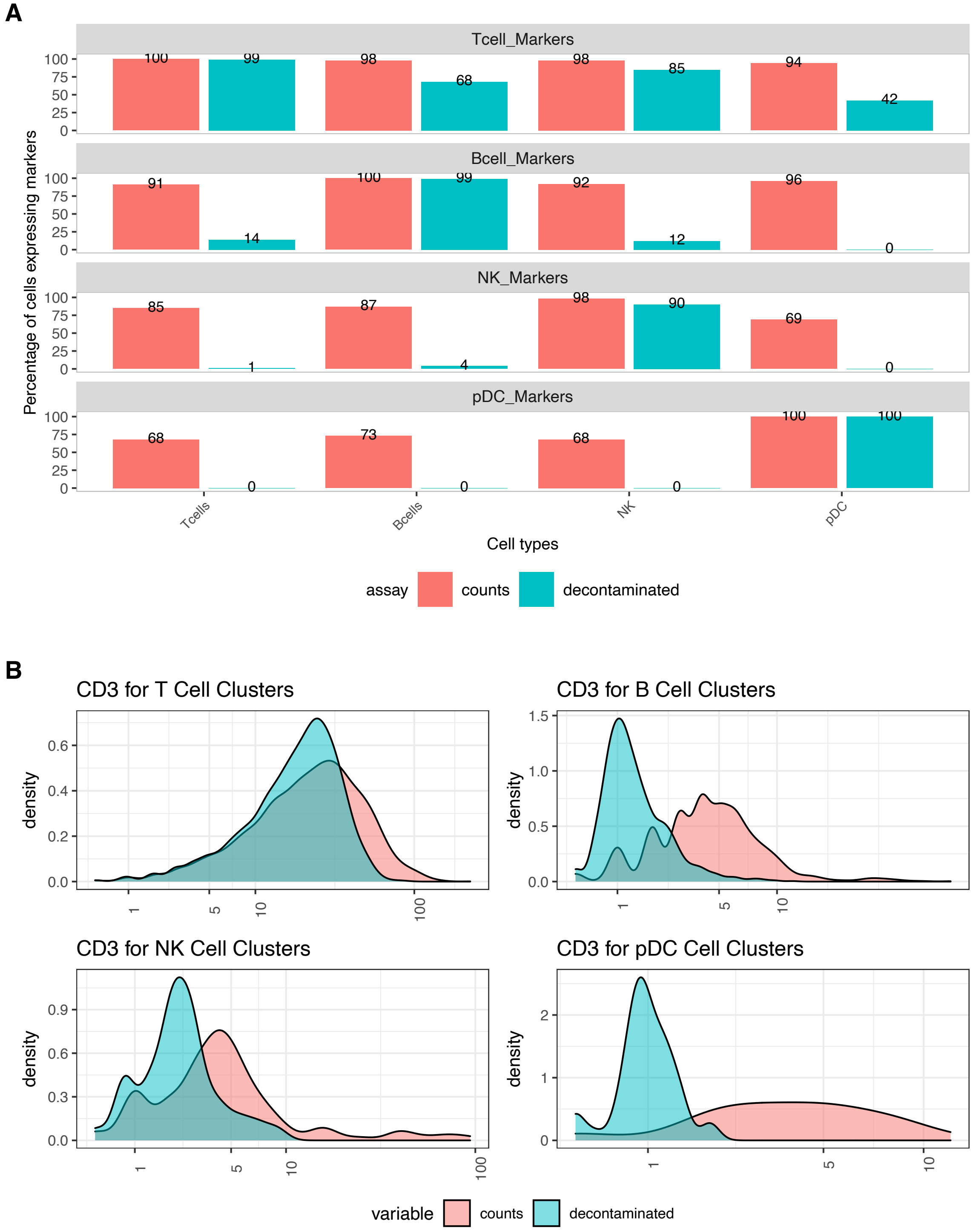

Figure S18. DecontPro applied to Leader dataset. A. Using the characteristic markers T cell: CD3; B cell: CD19; Natural Killer cell: CD56; plasmacytoid dendritic cells: CD123, markers are generally only preserved in native cell type clusters. **B.** Although the T-cell marker CD3 was still detected in non-native populations such as B-cells and pDCs, the expression of CD3 was largely reduced in all populations except for T cells.
